# Targeting kinesin family member 20A sensitizes stem-like triple-negative breast cancer cells to standard chemotherapy

**DOI:** 10.1172/JCI182394

**Published:** 2025-12-15

**Authors:** Yayoi Adachi, Weilong Chen, Cheng Zhang, Tao Wang, Nina Gildor, Rachel Shi, Haoyong Fu, Masashi Takeda, Qian Liang, Fangzhou Zhao, Hongyi Liu, Jun Fang, Jin Zhou, Hongwei Yao, Lianxin Hu, Shina Li, Lei Guo, Lin Xu, Ling Xie, Xian Chen, Chengheng Liao, Qing Zhang

**Affiliations:** 1 Department of Pathology, University of Texas Southwestern Medical Center, Dallas, Texas, USA.; 2 Department of Breast and Endocrine Surgery, Nagoya University Graduate School of Medicine, Nagoya, Aichi, Japan.; 3 Jinfeng Laboratory, Chongqing, China.; 4 Quantitative Biomedical Research Center, Peter O’Donnell Jr. School of Public Health, University of Texas Southwestern Medical Center, Dallas, Texas, USA.; 5 Department of Biochemistry and Biophysics, University of North Carolina, Chapel Hill, North Carolina, USA.; 6 Simmons Comprehensive Cancer Center, University of Texas Southwestern Medical Center, Dallas, Texas, USA.

**Keywords:** Cell biology, Oncology, Breast cancer, Stem Cells

## Abstract

Triple-negative breast cancer (TNBC), being both aggressive and highly lethal, poses a major clinical challenge in terms of treatment. Its heterogeneity and lack of hormone receptors or HER2 expression further restrict the availability of targeted therapy. Breast cancer stem cells (BCSCs), known to fuel TNBC malignancy, are now being exploited as a vulnerability for TNBC treatment. Here, we dissected the transcriptome of BCSCs and identified kinesin family member 20A (KIF20A) as a key regulator of BCSC survival and TNBC tumorigenesis. Genetic depletion or pharmacological inhibition of KIF20A impairs BCSC viability and tumor initiation and development in vitro and in vivo. Mechanistically, KIF20A supports BCSC stemness through modulation of mitochondrial oxidative phosphorylation, which is repressed by SMARCA4, a component of the SWI/SNF chromatin remodeling complex. Therapeutically, KIF20A inhibition sensitizes TNBC xenografts to standard-of-care chemotherapy. Our study highlights the importance of targeting KIF20A to exploit BCSC vulnerabilities in TNBC.

## Abstract 2

KIF20A is essential for triple-negative breast cancer stem cells, and its inhibition suppresses tumor initiation and enhances chemotherapy response, offering a potential treatment strategy.

## Graphical abstract

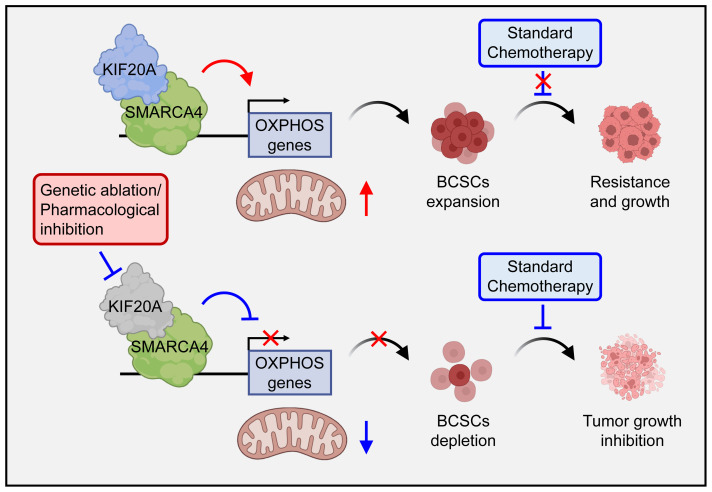

## Introduction

Triple-negative breast cancer (TNBC) is a distinct subtype lacking hormone receptors and human epidermal growth factor 2 (HER2), accounting for 15%–20% of all breast cancers (1–3). TNBC is the most aggressive subtype with early recurrence and metastasis (4, 5). Because of the absence of estrogen, progesterone, and HER2 receptors, TNBC does not respond to endocrine or HER2-targeted therapies, leaving chemotherapy and radiotherapy as the main treatments (6–9). Recently, anti–PD-1 or –PD-L1 immunotherapy has shown a limited objective response rate of no more than 10%, which is not as robust as in other solid tumors in clinical trials (10–12). Therefore, extensive efforts have focused on developing targeted therapies for TNBC, yet its pronounced heterogeneity remains a key challenge limiting therapeutic success (1, 13–17).

Breast cancer stem cells (BCSCs) are a subset of breast cancer cells with stem cell–like properties that drive tumor initiation, growth, and heterogeneity (10–12). BCSCs are also associated with resistance to chemotherapy and radiotherapy (13). Notably, TNBC has been identified as being enriched for BCSCs, contributing to their heterogeneity and making treatment challenging (14). Therefore, targeting BCSCs is crucial to improve TNBC prognosis. However, there is currently a lack of established therapies targeting BCSCs (15).

In this study, we used fluorescence-activated cell sorting (FACS) with multiple cancer stem cell markers to isolate BCSC subpopulations for transcriptomic analysis and identify key regulators controlling BCSCs in TNBC. We identified kinesin family member 20A (KIF20A) as an important factor in the control of BCSC properties by formation of a complex with the SWI/SNF complex protein SMARCA4, controlling gene expression important in the oxidative phosphorylation pathway in TNBC. Notably, pharmacologic inhibition of KIF20A, which reduces BCSC fitness, sensitized TNBC tumor models to standard chemotherapy. This study investigates the mechanism by which KIF20A regulates BCSCs and evaluates its potential as a novel therapeutic target for TNBC.

## Results

### Screening for potential determinants of breast cancer stem-like cells.

**Figure 1:**
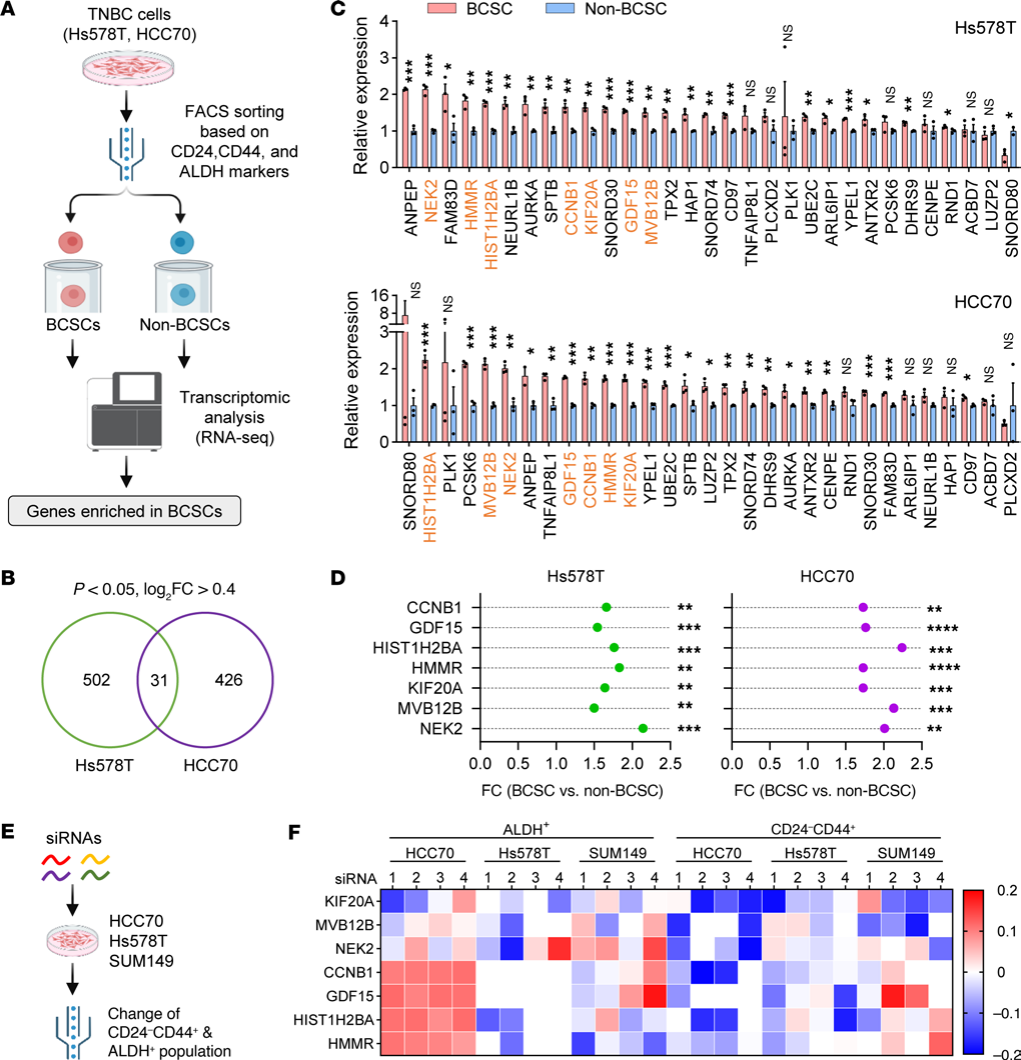
Identification of key regulators in breast cancer stem cells. (**A**) Experimental design of a screening strategy to identify critical genes essential for breast cancer stem cells (BCSCs). FACS, fluorescence-activated cell sorting. (**B**) Venn diagram of top differentially expressed genes (DEGs) in BCSCs versus non-BCSCs with indicated criteria and overlapped between 2 cell lines. (**C**) RT-qPCR analysis of the overlapped DEGs expressed in BCSCs and non-BCSCs derived from Hs578T and HCC70 cells (*n* = 3). Those marked in bold are overlapped genes significantly enriched in both Hs578T and HCC70 cells from RT-qPCR results. (**D**) Fold changes (FC) and *P* values are shown for top candidates in **C**. (**E**) Study design of siRNA-based gene knockdown and functional validation in BCSC populations. (**F**) Relative fold changes of CD24^–^CD44^+^ and ALDH^+^ populations measured by flow cytometry after knockdown of each candidate gene using 4 individual siRNAs (1 to 4) separately in indicated cell lines. Results are shown as fold changes over control siRNA. Data represent mean ± SEM. Statistical analyses were conducted by 2-tailed Student’s *t* test. *P < 0.05, **P < 0.01, ***P < 0.001, ****P < 0.0001.

BCSCs are regarded as tumor-initiating drivers that promote tumor progression and malignancy (13). Targeting BCSCs represents a promising strategy to suppress tumor initiation and overcome therapeutic resistance in breast cancer (16). Previous studies have shown that Ras/MARK, Wnt, TGF-β, Hedgehog, and Notch pathways maintain BCSC survival (15). However, there are no established target therapies for BCSCs, and potential regulators controlling BCSCs in TNBC remain understudied (15). BCSCs are commonly marked by CD24^–^CD44^+^ or ALDH^+^ phenotypes, representing invasive mesenchymal-like and proliferative epithelial-like subpopulations, respectively (17, 18). To identify key regulators differentially expressed in BCSCs versus non-BCSCs, we isolated BCSCs marked with CD24^–^CD44^+^ and ALDH^+^ using FACS from Hs578T and HCC70 cell lines, which exhibited moderate proportions of both populations suitable for sorting (Supplemental Figure 1, A–C; supplemental material available online with this article; https://doi.org/10.1172/JCI182394DS1). Cells lacking both markers (non-CD24^–^CD44^+^ and ALDH^–^) were defined as non-BCSCs (Supplemental Figure 2). We then performed transcriptomic profiling by RNA sequencing (RNA-Seq) on BCSCs and non-BCSCs from both cell lines (Figure 1A). Differentially expressed genes (DEGs) (BCSCs vs. non-BCSCs) were filtered with the following criteria: (a) significance defined by *P* value (*P* < 0.05) and (b) fold changes (log_2_ fold change > 0.4). We identified 533 and 457 DEGs in Hs578T and HCC70 cells, respectively (Supplemental Table 1). Among the DEGs, 31 genes showed consistent differential expression across both cell lines (Figure 1B). We performed quantitative reverse transcription PCR (RT-qPCR) to validate the expression of these 31 genes in sorted BCSCs and non-BCSCs from Hs578T and HCC70 cells (Figure 1C). Among them, 7 genes — *CCNB1*, *GDF15*, *HIST1H2BA*, *HMMR*, *KIF20A*, *MVB12B*, and *NEK2* — were significantly upregulated in BCSCs from both TNBC lines (Figure 1D). To assess whether these genes regulate BCSC abundance, we transfected each of the 7 genes with 4 individual siRNAs into 3 TNBC cell lines (HCC70, Hs578T, and SUM149). We then conducted flow cytometry to measure the CD24^–^CD44^+^ and ALDH^+^ BCSCs populations upon knockdown of each gene (Figure 1E). Our results showed that KIF20A knockdown consistently reduced both CD24^–^CD44^+^ and ALDH^+^ BCSC populations across all cell lines (Figure 1F). The role of KIF20A in regulating BCSCs remains undefined, which prompted us to investigate its function in TNBC stemness.

### KIF20A depletion decreases BCSC populations and related markers.

**Figure 2:**
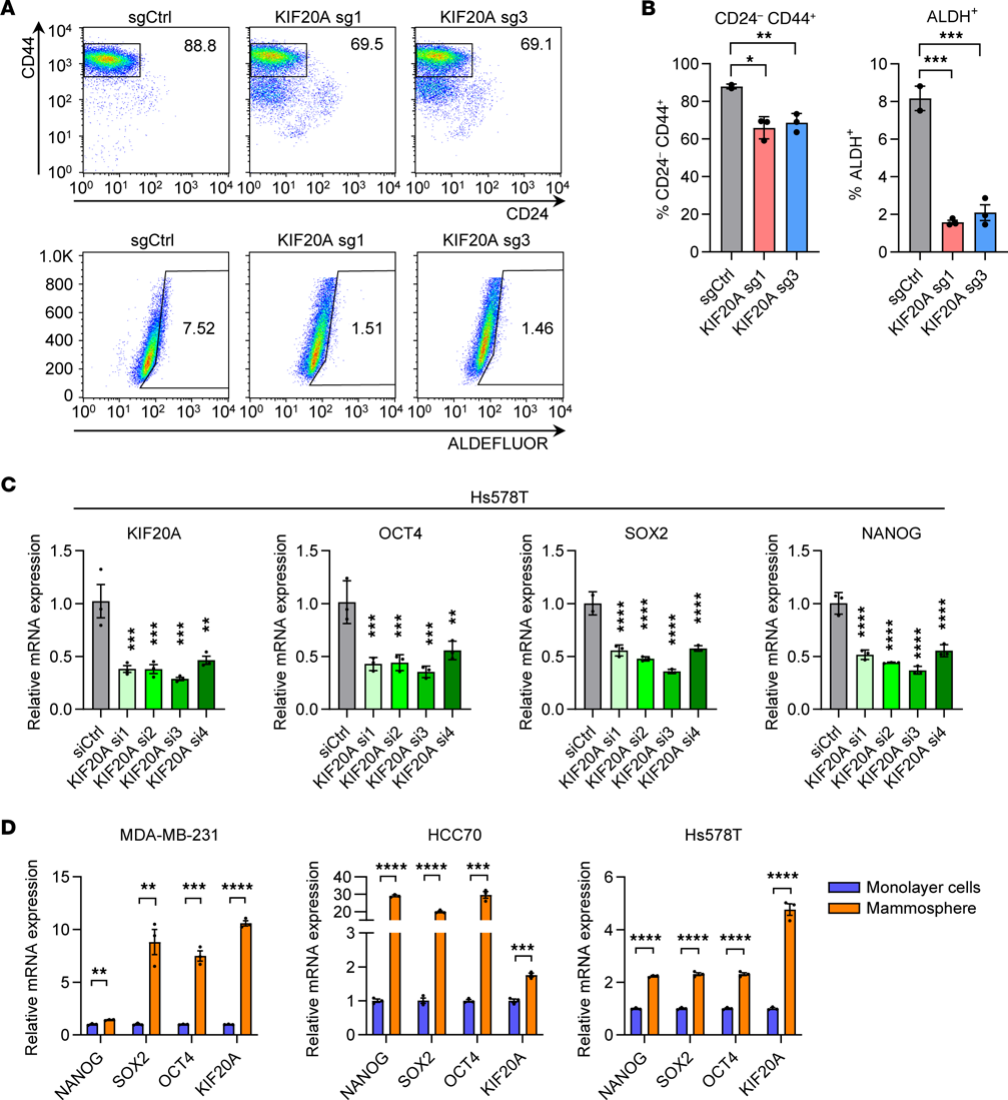
KIF20A depletion decreases BCSC populations and related markers. (**A** and **B**) Flow cytometry analysis for CD24^–^CD44^+^ and ALDH^+^ populations in TNBC stable cells infected with control or KIF20A sgRNA lentivirus. Representative images of flow cytometry (**A**) and quantification of CD24^–^CD44^+^ and ALDH^+^ cells (**B**) are shown. *n* = 3. (**C**) RT-qPCR analysis of indicated CSC markers and KIF20A gene in Hs578T cells with KIF20A knockdown by individual siRNAs. *n* = 3. (**D**) RT-qPCR analysis of indicated CSC markers and KIF20A gene in indicated TNBC parental cells and mammospheres derived from the mammosphere formation assay. *n* = 3. Each data point represents a biological replicate. Data represent mean ± SEM. Statistical analyses were conducted by 1-way ANOVA with Dunnett’s test (**B** and **C**) or 2-tailed Student’s *t* test (**D**). *P < 0.05, **P < 0.01, ***P < 0.001, ****P < 0.0001.

To further validate whether KIF20A depletion reduces BCSC populations, we generated 2 independent sgRNAs against KIF20A. In MDA-MB-231 cells, KIF20A depletion markedly decreased both CD24^–^CD44^+^ and ALDH^+^ populations (Figure 2, A and B). To confirm that KIF20A regulates breast cancer stemness, we assessed the expression of canonical stem cell markers (Oct4, Sox2, or NANOG) and found that KIF20A siRNA transfection led to decreased mRNA levels of these markers in Hs578T cells (Figure 2C). We next compared KIF20A expression between 2D monolayer cultures and BCSC-enriched mammospheres derived from the mammosphere formation assay, a well-established method for evaluating the BCSC populations (19). KIF20A expression was significantly elevated in mammospheres from multiple TNBC cell lines compared with their parental 2D cultures (Figure 2D), consistent with our finding in Figure 1C. Accordingly, *Oct4*, *Sox2*, and *NANOG* gene expression was induced in mammospheres compared with parental cells (Figure 2D).

### KIF20A controls self-renewal and population expansion of BCSCs in vitro.

**Figure 3:**
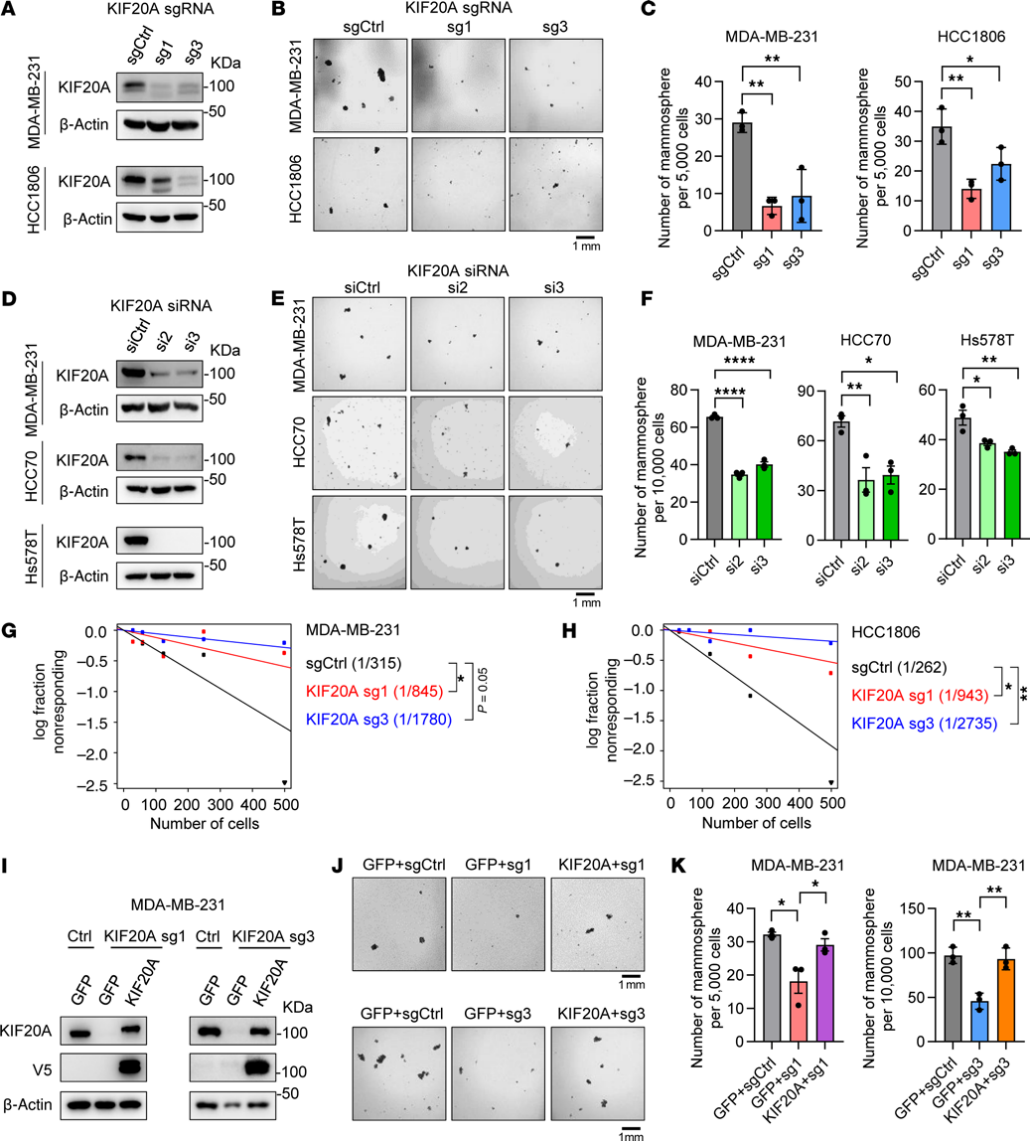
KIF20A depletion decreases BCSC self-renewal and expansion in vitro. (**A**–**C**) Immunoblot analysis of KIF20A (**A**), representative images of mammosphere formation assay (**B**), and corresponding quantifications (**C**) in MDA-MB-231 and HCC1806 cells infected with lentivirus encoding control or KIF20A sgRNAs. *n* = 3. Scale bar: 1 mm. (**D**–**F**) Immunoblot analysis of KIF20A (**D**), representative images of mammosphere formation assay (**E**), and quantification of mammospheres (**F**) in indicated cells transfected with control or KIF20A siRNAs. *n* = 3. Scale bar: 1 mm. (**G** and **H**) Extreme limiting dilution assays (**G**) and calculated estimated BCSC frequency (**H**) in MDA-MB-231 and HCC1806 cells infected with control or KIF20A sgRNA lentivirus. (**I**–**K**) Immunoblot analysis (**I**), mammosphere formation assay (**J**), and quantification of mammospheres (**K**) of GFP- or KIF20A-expressing MDA-MB-231 stable cells infected with virus encoding control or KIF20A sgRNAs. *n* = 3. Scale bar: 1 mm. Each data point represents a biological replicate. Data represent mean ± SEM. Statistical analyses were conducted by 1-way ANOVA with Dunnett’s test (**C** and **F**) or Tukey’s test (**K**), or χ^2^ test (**G**). *P < 0.05, **P < 0.01, ****P < 0.0001.

To validate the role of KIF20A in regulating BCSCs in TNBC, we depleted KIF20A using sgRNAs in MDA-MB-231 and HCC1806 TNBC cells (Figure 3A). Since MVB12B also emerged as a candidate from our initial screen (Figure 1F), we also generated 3 sgRNAs that effectively target MVB12B in MDA-MB-231 and Hs578T cells (Supplemental Figure 3, A and B). Next, equal numbers of control and sgRNA-depleted cells were plated for mammosphere formation assays (19) to assess self-renewal capacity. KIF20A depletion significantly reduced mammosphere formation in both TNBC cell lines (Figure 3, B and C), whereas MVB12B depletion had no significant effect (Supplemental Figure 3, C–F). To further validate these findings, we generated shRNAs (#1, #2) in stable cells or transiently transfected TNBC cells with 2 independent KIF20A siRNAs (#2, #3). Consistent with sgRNA results, KIF20A knockdown by shRNAs or siRNAs decreased mammosphere formation (Figure 3, D–F, and Supplemental Figure 3, G–I). These findings demonstrate that KIF20A is critical for BCSC self-renewal and expansion in vitro.

KIF20A, a member of the kinesin-6 family within the kinesin superfamily of proteins (KIFs), functions as a molecular motor that transports cellular cargo and plays an essential role in cell division (20, 21). The attenuation of BCSC self-renewal upon KIF20A depletion prompted us to assess its effect on cancer stem cell (CSC) frequency using the extreme limiting dilution assay (22–26). In MDA-MB-231 cells, the estimated stem cell frequency was 1 in 315 with control sgRNA, which decreased to 1 in 845 and 1 in 1,780 upon infection with KIF20A sgRNA #1 and sgRNA #3, respectively. Similar results were observed in HCC1806 cells (the frequency dropped from 1 in 262 in controls to 1 in 943 and 1 in 2,735 with KIF20A sg1 and sg3, respectively) (Figure 3, G and H).

To confirm that these phenotypes resulted from on-target effects, we generated sgRNA-resistant KIF20A expression constructs by introducing silent mutations within the CRISPR/Cas9 NGG recognition sites for sgRNAs #1 and #3. These constructs were introduced into KIF20A-depleted TNBC cells via viral infection. As expected, expression of the resistant constructs restored KIF20A protein levels to those comparable to control cells (Figure 3I). Importantly, reintroduction of sgRNA-resistant KIF20A rescued the mammosphere formation defect caused by KIF20A depletion (Figure 3, J and K). Collectively, these results establish that KIF20A is essential for maintaining BCSC self-renewal and population expansion in TNBC.

### KIF20A is essential to BCSC survival in vivo and TNBC tumor development.

**Figure 4:**
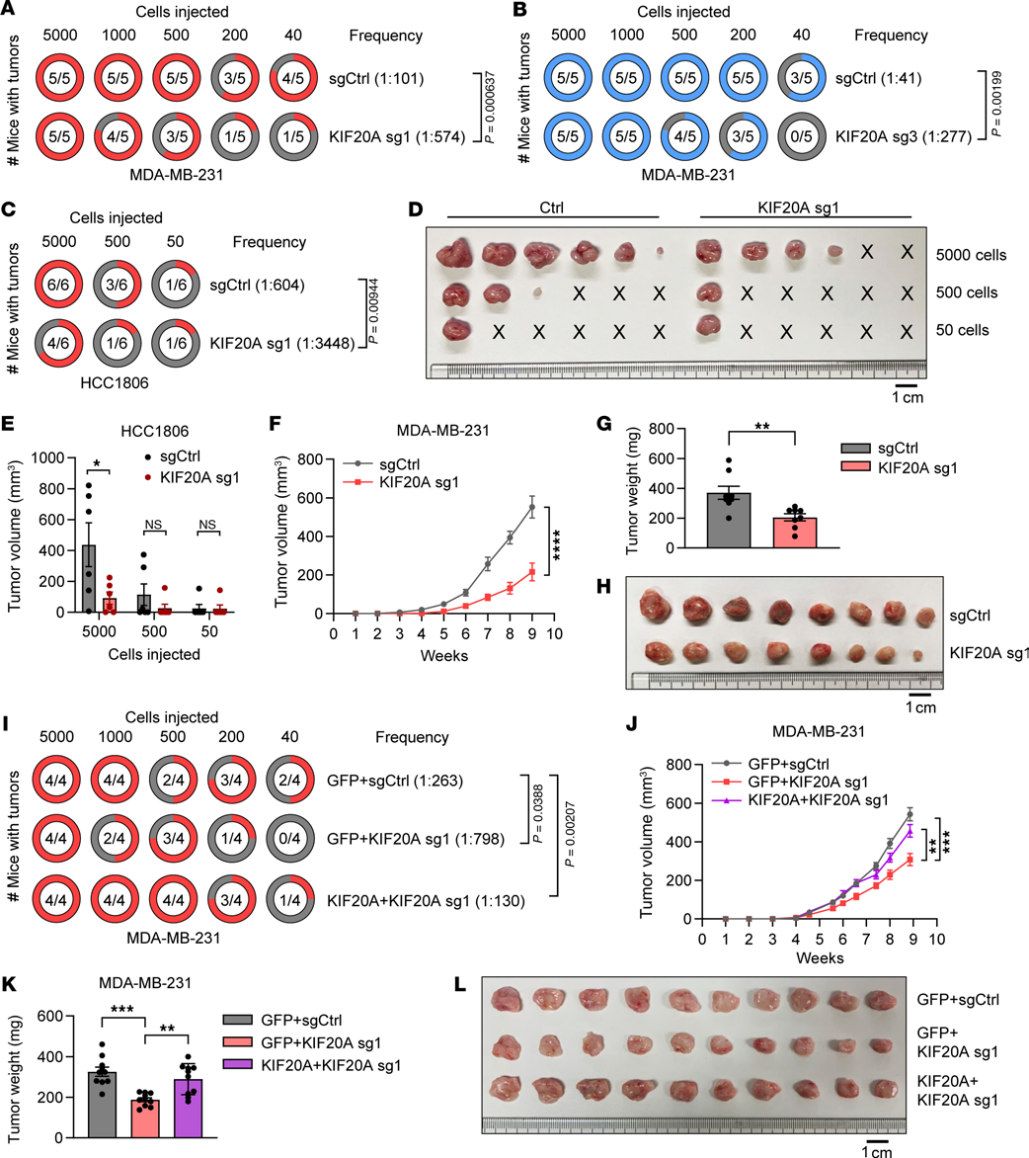
KIF20A depletion decreases BCSC self-renewal and TNBC tumorigenesis in vivo. (**A** and **B**) Limiting dilution tumor-initiating assay of MDA-MB-231 cells infected with lentivirus encoding control or KIF20A sgRNA #1 or #3. *n* = 5 mice for each group. (**C**–**E**) Limiting dilution tumor-initiating assay (**C**), harvested tumor images (**D**), and tumor volume at the end point (**E**) in HCC1806 cells infected with lentivirus encoding control or KIF20A sgRNA #1. *n* = 6 mice for each group. (**F**–**H**) Tumor growth (**F**), tumor weight (**G**), and tumor images after sacrifice (**H**) of MDA-MB-231 stable cells infected with lentivirus encoding control or KIF20A sgRNA #1 injected into mammary glands of NSG mice. (**I**) Limiting dilution tumor-initiating assay of MDA-MB-231 cells expressing control or KIF20A sgRNA and GFP or sgRNA-resistant KIF20A mutant as indicated. (**J**–**L**) Tumor growth (**J**), tumor weight (**K**), and tumor images after sacrifice (**L**) of MDA-MB-231 stable cells expressing control or KIF20A sgRNA and GFP or sgRNA-resistant KIF20A mutant as indicated in NSG mice. Each data point represents a biological replicate. Data represent mean ± SEM. Statistical analyses were conducted by χ^2^ test (**A**–**C** and **I**), 2-tailed Student’s *t* test (**E** and **G**), 2-way ANOVA (**F** and **J**), or 1-way ANOVA with Tukey’s test (**K**). *P < 0.05, **P < 0.01, ***P < 0.001, ****P < 0.0001. Scale bars: 1 cm.

To determine whether KIF20A regulates BCSC activity to promote TNBC tumor initiation in vivo, we performed limiting dilution assays in NOD-scid IL-2Rγ–null (NSG) mice. The tumor initiation assay with limiting dilution is widely recognized as a rigorous method for evaluating the impact of specific factors on CSC frequency in vivo (24, 25). We performed 2 independent experiments with 2 different KIF20A sgRNAs (sg1, sg3) in MDA-MB-231 cells. Control or KIF20A-depleted cells were orthotopically injected into the fourth mammary fat pad of female NSG mice with 5 serial dilutions (5,000, 1,000, 500, 200, and 40 cells). After implantation, tumor incidence was recorded, and CSC frequency was calculated accordingly. KIF20A depletion significantly decreased tumor incidence with decreased CSC frequency for sg1 (1:574 in KIF20A-KO vs. 1:101 in control group, *P* = 0.000637) (Figure 4A), and a similar result was obtained for sg3 (1:277 in KIF20A-KO vs. 1:41 in control group, *P* = 0.00199) (Figure 4B). Similarly, depletion of KIF20A in another TNBC line, HCC1806, led to a reduction in CSC frequency (1:3,448 in KIF20A-KO vs. 1:604 in control group, *P* = 0.00944) (Figure 4C), accompanied by smaller tumor volumes especially in the 5,000 dilution group (Figure 4, D and E). Minor variations observed among control sgRNA groups likely reflect biological variability between independent experiments. Our results suggest that KIF20A is essential for maintaining BCSC population in vivo. Consistently, KIF20A depletion also resulted in impaired TNBC tumor growth, which resulted in reduced tumor weight and smaller size of xenografts at necropsy (Figure 4, F–H). Furthermore, KIF20A-depleted tumors, which were verified by Western blot (Supplemental Figure 4A), showed reduced expression of *Oct4*, *Sox2*, and *NANOG*, underpinning that the BCSC population was decreased in these tumors (Supplemental Figure 4B). Given that tumor progression is not solely driven by CSCs and that non-CSCs can also promote tumor progression (27), we investigated the effect of KIF20A on the proliferation of non-BCSCs. We sorted non-BCSCs using FACS from the siRNA control Hs578T cells and siRNA-mediated KIF20A-downregulated cells (Supplemental Figure 4C) and immediately performed 2D colony formation assay and MTS assay. We found that cell growth in these non-BCSCs was slightly decreased by KIF20A knockdown (Supplemental Figure 4, D and E).

To confirm the on-target effect of KIF20A sgRNAs on BCSC initiation in vivo, we carried out tumor initiating assay with limiting dilutions by re-expressing sgRNA-resistant KIF20A in KIF20A-depleted cells (sg1). We found that sgRNA-resistant KIF20A expression could fully rescue the impairment of BCSC frequency caused by KIF20A deletion, arguing that the effect of KIF20A sgRNA on BCSC initiation was due to its on-target depletion of KIF20A protein levels in TNBC (Figure 4I). Consistently, re-expression of sgRNA-resistant KIF20A also rescued tumor growth defects caused by KIF20A depletion (Figure 4, J–L). Together, our results show that KIF20A is essential for TNBC tumor growth through controlling BCSC fate.

To examine whether KIF20A is sufficient to drive BCSC expansion and tumor progression, we overexpressed KIF20A in MDA-MB-231 and HCC1806 cells (Supplemental Figure 5A). However, KIF20A overexpression did not significantly alter mammosphere formation in vitro (Supplemental Figure 5, B and C). To confirm this in vivo, we generated doxycycline-inducible (Dox-inducible) KIF20A or empty vector control cells (Supplemental Figure 5D) and performed tumor initiation and growth assays in mice fed with Dox food. Similarly, KIF20A overexpression did not change the CSC frequency in vivo (Supplemental Figure 5E) or tumor growth (Supplemental Figure 5, F–H). These results suggest that KIF20A may be essential but not sufficient for BCSC expansion or tumor progression. Therefore, depletion or inhibition of KIF20A could be therapeutically beneficial in TNBC.

### Pharmacological inhibition of KIF20A decreases BCSCs and suppresses TNBC tumor growth.

**Figure 5:**
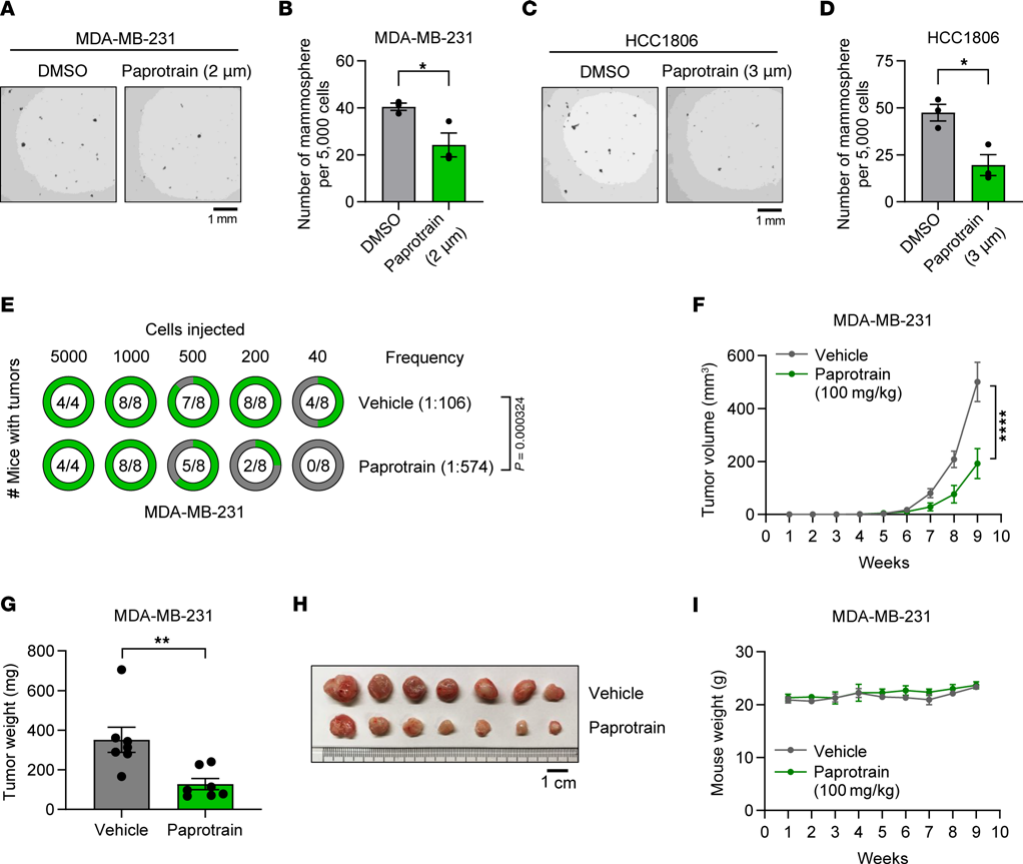
KIF20A inhibitor decreases BCSC self-renewal ability and expansion in vivo. (**A** and **B**) Mammosphere formation assays with representative images (**A**) and corresponding quantification (**B**) in MDA-MB-231 cells treated with 2 μM of paprotrain. *n* = 3. (**C** and **D**) Mammosphere formation assay with representative images (**C**) and corresponding quantification (**D**) in HCC1806 cells treated with 3 μM of paprotrain. *n* = 3. Scale bars: 1 mm. (**E**) Limiting dilution tumor-initiating assay with MDA-MB-231 cells treated with vehicle or paprotrain (100 mg/kg/wk). (**F**–**I**) Tumor growth (**F**), tumor weight after dissection (**G**), image of tumors (**H**), and body weight (**I**) of MDA-MB-231 xenograft tumors treated with vehicle or paprotrain (100 mg/kg/wk). *n* = 7 mice for each group. Scale bar: 1 cm. Each data point represents a biological replicate. Data represent mean ± SEM. Statistical analyses were conducted by χ^2^ test (**E**), 2-tailed Student’s *t* test (**B**, **D**, and **G**), or 2-way ANOVA (**F**). *P < 0.05, **P < 0.01, ****P < 0.0001.

To validate the impact of genetic ablation of KIF20A on BCSCs in TNBC, we investigated the potential effect of pharmacological inhibition of KIF20A. To achieve this, we used a characterized KIF20A inhibitor, paprotrain (Supplemental Figure 6A), which inhibits ATPase activity of KIF20A with an IC_50_ of 1.35 μM (28). A previous study demonstrated that depletion of KIF20A through RNAi led to an increase of binucleated cells (28). To validate the on-target inhibition of KIF20A by paprotrain, we quantified binucleated cells across a range of on-target concentrations (0.5–4 μM). Our results showed that paprotrain treatment led to significantly increased binucleation of TNBC cells in a dose-dependent manner (Supplemental Figure 6, B and C). We then examined the effect of paprotrain on the BCSC population using the mammosphere formation assay. Our results showed that paprotrain treatment, especially at the dosage (2 μM in MDA-MB-231 and 3 μM in HCC1806 cells) that caused significant KIF20A activity inhibition (Supplemental Figure 6, B and C), led to decreased mammosphere formation in these cells (Figure 5, A–D). Because mammosphere culture extends over 2 weeks and fresh compound cannot be replenished, we applied slightly higher yet still on-target concentrations to ensure sustained inhibition. To evaluate whether paprotrain suppresses BCSC activity in vivo, we implanted MDA-MB-231 cells orthotopically in female NSG mice. One week after inoculation, we administered paprotrain intraperitoneally once per week (100 mg/kg), and monitored tumor initiation and tumor size. Paprotrain administration significantly decreased tumor initiation with decreased CSC frequency (1/459 in paprotrain vs. 1/106 in control group, *P* = 0.000324) (Figure 5E). Furthermore, paprotrain treatment led to decreased tumor growth, which corresponded to decreased tumor weight and size at necropsy (Figure 5, F–H). Importantly, paprotrain treatment did not lead to significant change in mouse body weight, suggesting no overt toxicity (Figure 5I). Collectively, we confirmed the essential role of KIF20A in sustaining the ability of BCSCs in TNBC through multipronged approaches.

### KIF20A regulates OXPHOS-related gene expression in TNBC.

**Figure 6:**
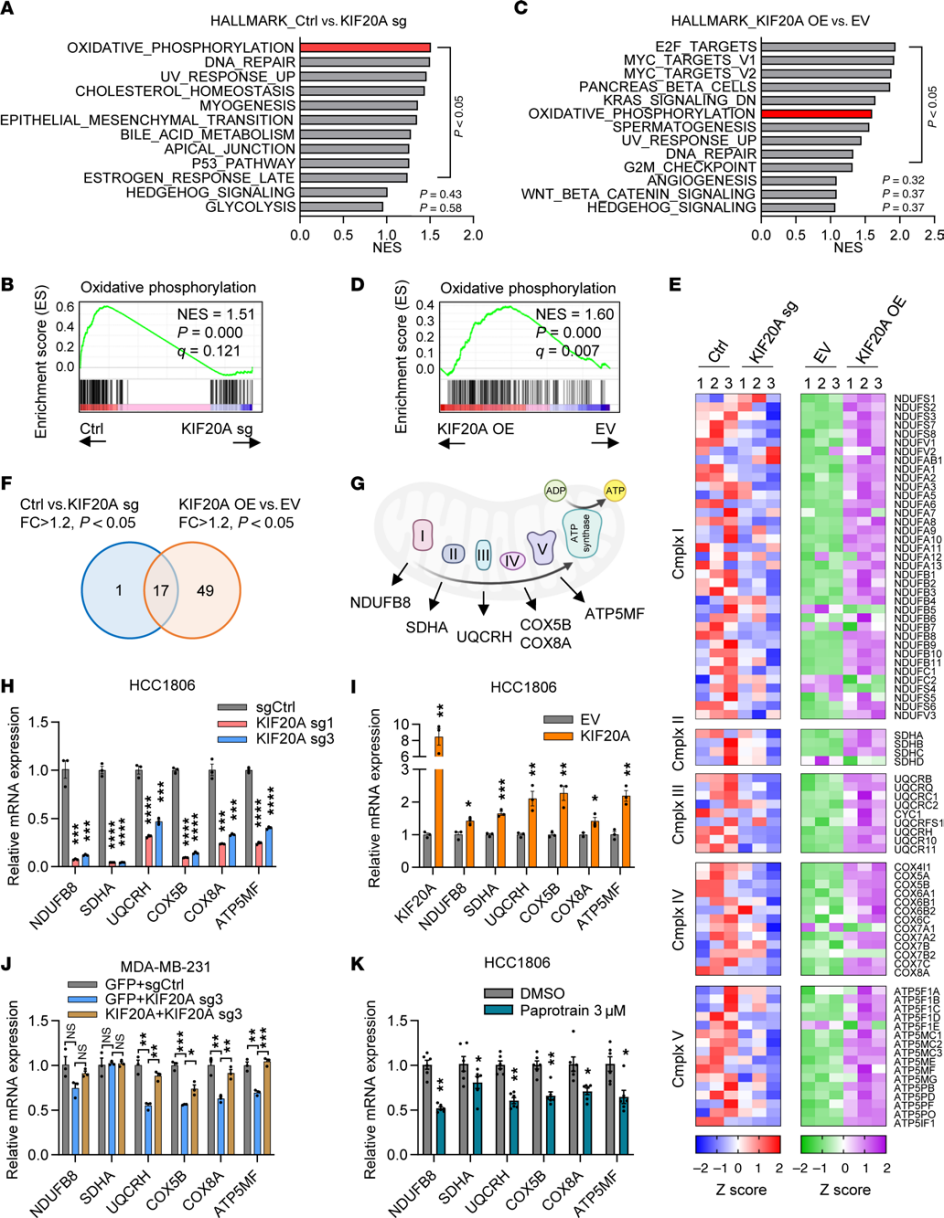
KIF20A affects the expression of mitochondrial OXPHOS genes. (**A**–**D**) Gene set enrichment analysis (GSEA) showing the top hallmark pathways enriched in the control sgRNA compared with KIF20A sg1 (**A**) or KIF20A overexpression compared with the EV control (**C**) in MDA-MB-231 cells. GSEA enrichment plots of the OXPHOS pathways from KIF20A knockdown (**B**) and KIF20A overexpression (**D**) are shown. (**E**) Heatmap of genes related to mitochondrial complex I–V in control or KIF20A-depleted and -overexpressing MDA-MB-231 cells from the RNA-Seq data. *n* = 3. (**F**) Venn diagram of significant genes associated with mitochondrial complexes identified from the KIF20A-depleted and -overexpressing RNA-Seq experiments. (**G**) Representative genes selected for functional validation from each mitochondrial complex. (**H**) RT-qPCR analysis of selected OXPHOS genes in KIF20A-depleted HCC1806 cells. *n* = 3. (**I**) RT-qPCR analysis of OXPHOS genes in HCC1806 cells with KIF20A overexpression. *n* = 3. (**J**) RT-qPCR analysis of OXPHOS genes in KIF20A cells rescued by sgRNA-resistant KIF20A mutant in MDA-MB-231 cells. *n* = 3. (**K**) RT-qPCR analysis of OXPHOS genes in HCC1806 cells treated with paprotrain. *n* = 6. Each data point represents a biological replicate. Data represent mean ± SEM. Statistical analyses were conducted by 1-way ANOVA with Dunnett’s test (**H**), 2-tailed Student’s *t* test (**I** and **K**), or 1-way ANOVA with Tukey’s test (**J**). *P < 0.05, **P < 0.01, ***P < 0.001, ****P < 0.0001.

To understand the molecular mechanism by which KIF20A regulates BCSCs in TNBC, we analyzed transcriptomic changes following *KIF20A* gene manipulation with 2 complementary strategies. First, we performed RNA-Seq with MDA-MB-231 cells with KIF20A depletion using the validated sgRNA #1. Conversely, we also conducted RNA-Seq with MDA-MB-231 cells overexpressing KIF20A. Gene set enrichment analysis (GSEA) revealed that oxidative phosphorylation (OXPHOS) was the top downregulated pathway in KIF20A-depleted cells (Figure 6, A and B), whereas it was significantly enriched in KIF20A-overexpressing cells compared with control cells (Figure 6, C and D). Although the metabolic phenotype of CSCs varies across cancer types and microenvironments, OXPHOS has been widely recognized as a major energy source supporting CSC maintenance (29–31). A previous study showed that the master mitochondrial biogenesis regulator peroxisome proliferator–activated receptor-γ coactivator 1α (PGC1α) maintains stemness characteristics in breast cancer (29). Another prior study demonstrated that metformin, an inhibitor of OXPHOS, reduces the size and number of mammospheres, as well as *OCT4*, a maker of BCSCs, in breast cancer (32). In contrast, canonical Wnt/β-catenin and Hedgehog signaling pathways, both previously implicated in BCSC regulation (33), did not reach statistical significance in our GSEA for either KIF20A depletion or overexpression, suggesting that KIF20A may regulate BCSCs through Wnt/β-catenin– and Hedgehog-independent signaling in TNBC. Therefore, we decided to focus on OXPHOS as a potential mechanism through which KIF20A regulates BCSCs in TNBC.

The OXPHOS system comprises 5 enzyme complexes (I–V). RNA-Seq analysis identified significantly overlapping KIF20A-dependent genes across all complexes except complex II (Figure 6E). Seventeen genes were commonly regulated by both KIF20A depletion and overexpression (fold change >1.2, *P* < 0.05) (Figure 6F and Supplemental Table 2). For further analysis, we selected *NDUFB8* from complex I, *UQCRH* from complex III, *COX5B* and *COX8A* from complex IV, and *ATP5MF* from complex V, as they were among the overlapping genes (Figure 6G). From complex II, *SDHA* was chosen because its expression was significantly induced by KIF20A overexpression and showed a tendency of downregulation by KIF20A depletion. Quantitative RT-PCR analysis confirmed that 5 of the 6 selected OXPHOS genes (excepting *SDHA*) were downregulated upon KIF20A depletion by 2 independent sgRNAs (#1, #3) in 3 TNBC cell lines (MDA-MB-231, HCC1806, and Hs578T) (Figure 6H and Supplemental Figure 7, A and B). Consistent results were obtained with shRNA-mediated knockdown in Hs578T cells (Supplemental Figure 7C). Conversely, overexpression of KIF20A upregulated these OXPHOS genes across all 3 TNBC cell lines (Figure 6I and Supplemental Figure 7, D and E). Importantly, re-expression of sgRNA-resistant KIF20A fully restored OXPHOS gene expression in KIF20A-depleted cells, confirming the on-target effect of KIF20A loss (Figure 6J). Pharmacological inhibition of KIF20A using paprotrain at physiologically relevant concentrations also reduced the expression of these OXPHOS genes (Figure 6K and Supplemental Figure 7F). Moreover, FACS-sorted BCSCs and non-BCSCs from MDA-MB-231 cells revealed that OXPHOS gene downregulation upon KIF20A depletion occurred specifically in the BCSC population but not in non-BCSCs (Supplemental Figure 7G). Together, our data suggest that KIF20A controls gene expression involved in OXPHOS, which may be important to regulate BCSCs in TNBC.

### KIF20A regulates mitochondrial OXPHOS important in maintaining BCSCs in TNBC.

**Figure 7:**
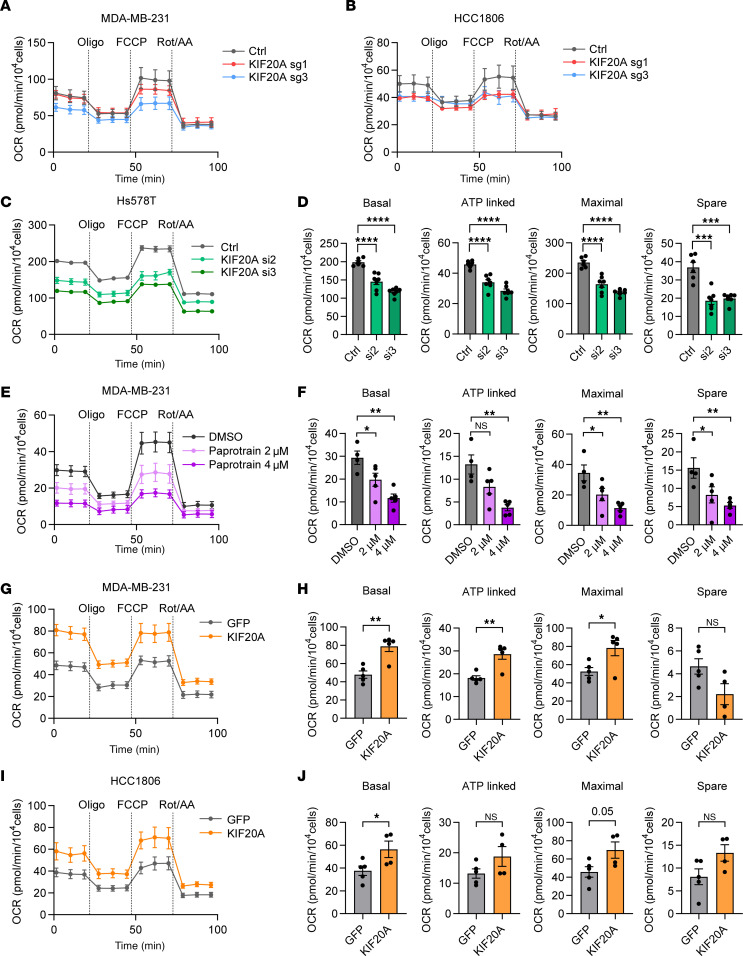
KIF20A controls mitochondrial OXPHOS. (**A** and **B**) Measurement of oxygen consumption rate (OCR) of MDA-MB-231 (**A**) and HCC1806 (**B**) cells expressing control or KIF20A sgRNAs. *n* = 5–7. (**C** and **D**) Measurement of OCR (**C**) and corresponding quantifications (**D**) in Hs578T cells transfected with control or KIF20A siRNAs. *n* = 6–7. (**E** and **F**) Measurement of OCR (**E**) and corresponding quantifications (**F**) in MDA-MB-231 cells treated with indicated doses of paprotrain. *n* = 4–5. (**G**–**J**) Measurement of OCR and corresponding quantifications in MDA-MB-231 (**G** and **H**) and HCC1806 (**I** and **J**) cells expressing GFP control or KIF20A. *n* = 4–5. Each data point represents a biological replicate. Data represent mean ± SEM. Statistical analyses were conducted by 1-way ANOVA with Dunnett’s test (**D** and **F**) or 2-tailed Student’s *t* test (**H** and **J**). *P < 0.05, **P < 0.01, ***P < 0.001, ****P < 0.0001.

To determine whether KIF20A controls mitochondrial function through regulating OXPHOS genes, we measured the oxygen consumption rate (OCR) in TNBC cells following KIF20A manipulation using Seahorse assays. First, KIF20A depletion by 2 independent sgRNAs (#1, #3) led to decreased OCR in MDA-MB-231 cells (Figure 7A) and HCC1806 cells (Figure 7B). Similarly, knockdown of KIF20A using 2 distinct siRNAs (#2, #3) decreased OCR in Hs578T cells (Figure 7, C and D). KIF20A inhibition with paprotrain treatment decreased OCR in a dose-dependent manner in MDA-MB-231 cells (Figure 7, E and F). Conversely, KIF20A overexpression resulted in significantly increased OCR in MDA-MB-231 (Figure 7, G and H) and HCC1806 cells (Figure 7, I and J).

To verify whether KIF20A modulates OCR through mitochondrial OXPHOS, we generated MDA-MB-231 cell lines depleted of mitochondrial DNA (mtDNA) using ethidium bromide (EtBr) (34). Treatment with EtBr for 1 week reduced the expression of the mtDNA-encoded genes *ND2* and *D-loop* at concentrations of 225 ng/mL or more (Supplemental Figure 8, A and B). MDA-MB-231 control or KIF20A-overexpressing cells were then cultured in either DMSO or EtBr (225 ng/mL) for 1 week to establish cell lines with normal or depleted mtDNA expression (Supplemental Figure 8, C and D). We performed Seahorse assays in these cell lines and found that the KIF20A-induced OCR was abolished by mtDNA depletion. Specifically, EtBr-treated cells showed no response to oligomycin, FCCP, or rotenone/antimycin A (Rot/AA), even with high KIF20A expression (Supplemental Figure 8, E and F). This suggests that the effect of KIF20A on OXPHOS relies on the integrity of the electron transport chain. To summarize, our results showed that KIF20A may regulate mitochondrial function in TNBC.

**Figure 8:**
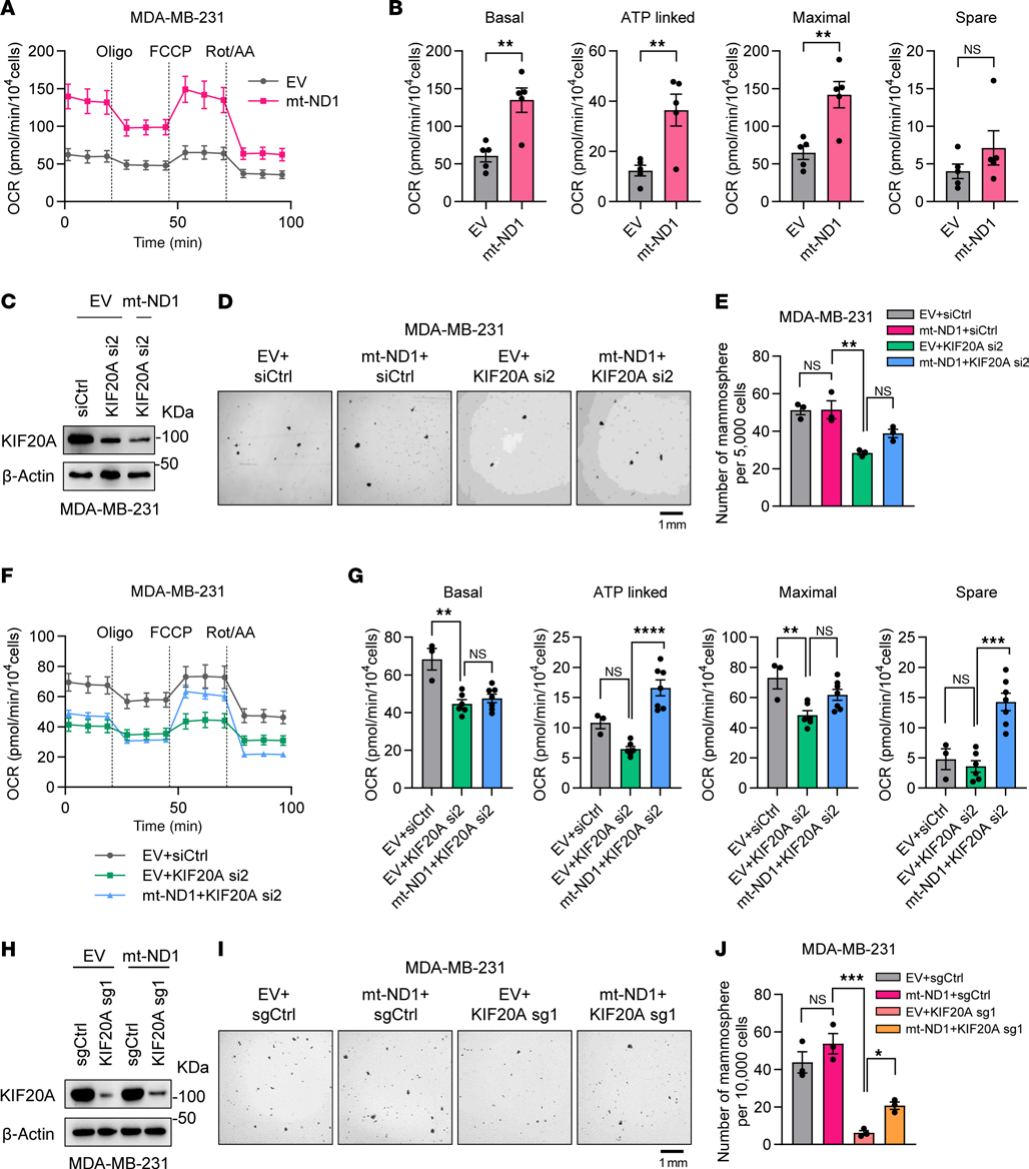
OXPHOS restoration by mt-ND1 rescues decreased BCSC self-renewal ability by KIF20A depletion. (**A** and **B**) Measurement of OCR (**A**) and corresponding quantifications (**B**) in MDA-MB-231 cells expressing empty vector (EV) or mt-ND1. *n* = 5. (**C**–**G**) Immunoblot analysis (**C**), representative images of mammosphere formation assay (**D**), corresponding mammosphere quantifications (*n* = 3) (**E**), and OCR measurement and quantifications (*n* = 3–7) (**F** and **G**) of KIF20A in EV- or mt-ND1–overexpressing MDA-MB-231 cells transfected with indicated control (Ctrl) or KIF20A siRNAs. Scale bar: 1 mm. (**H**–**J**) Immunoblot analysis of KIF20A (**H**), representative images of mammosphere formation assay (**I**), and corresponding mammosphere quantifications (*n* = 3) (**J**) in EV- or mt-ND1–overexpressing MDA-MB-231 cells transduced with control (Ctrl) or KIF20A sgRNA. Scale bar: 1 mm. Each data point represents a biological replicate. Data represent mean ± SEM. Statistical analyses were conducted by 2-tailed Student’s *t* test (**B**) or 1-way ANOVA with Tukey’s test (**E**, **G**, and **J**). *P < 0.05, **P < 0.01, ***P < 0.001, ****P < 0.0001.

The reduction in OCR upon KIF20A loss suggests impaired mitochondrial function. We next investigated whether restoring mitochondrial respiration could rescue the stem-like phenotype impaired by KIF20A depletion. We expressed the yeast mitochondrial NADH dehydrogenase NDI1 (hereafter referred to as mt-ND1) (Supplemental Figure 8G), which oxidizes NADH to NAD^+^ and compensates for mammalian complex I activity (35). First, mt-ND1 overexpression increased both basal and maximal respiration in MDA-MB-231 cells (Figure 8, A and B), confirming enhanced mitochondrial activity. Next, we performed mammosphere assays to examine the effect of mt-ND1 overexpression in TNBC cells depleted of KIF20A. Whereas KIF20A knockdown led to decreased mammosphere formation in TNBC, this phenotype could be partially rescued by mt-ND1 overexpression (Figure 8, C–E). Furthermore, mt-ND1 overexpression partially rescued OCR depletion caused by KIF20A depletion (Figure 8, F and G). In addition, we also performed rescue experiments in TNBC infected with the KIF20A sgRNA, and consistently, mt-ND1 overexpression rescued the phenotype of BCSCs upon KIF20A depletion (Figure 8, H–J). Cumulatively, our results suggest that KIF20A sustains BCSCs in TNBC through mitochondrial OXPHOS.

### KIF20A controls OXPHOS and BCSCs through SMARCA4.

**Figure 9:**
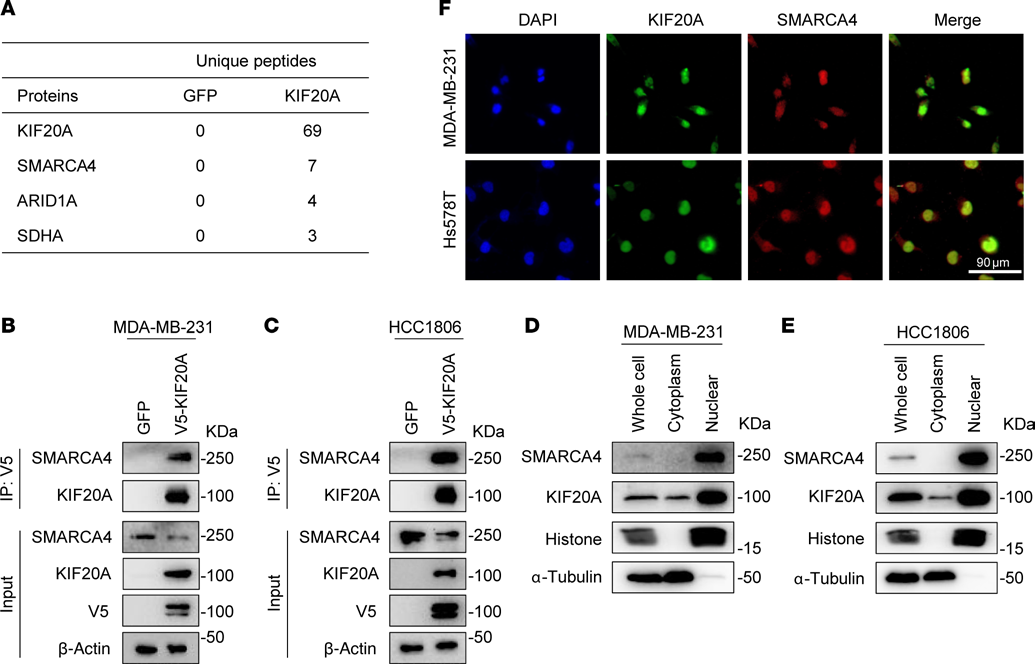
KIF20A interacts with SMARCA4. (**A**) Top hits of KIF20A binding proteins identified by immunoprecipitation–mass spectrometry. (**B** and **C**) Co-IP of exogenous HA-tagged KIF20A and endogenous SMARCA4 in MDA-MB-231 (**B**) or HCC1806 (**C**) cells. (**D** and **E**) Immunoblot analysis of extracted nuclear and cytoplasmic proteins in MDA-MB-231 (**D**) or HCC1806 (**E**) cells. (**F**) Immunofluorescence staining of endogenous KIF20A and SMARCA4 in MDA-MB-231 or Hs587T cell lines. Scale bar: 90 μm.

As a kinesin family member, KIF20A canonically mediates chromosome transport during mitosis via its ATPase-containing kinesin domain (20). Since KIF20A is not a transcription factor, how it regulates gene expression involved in mitochondrial function remains unclear. To identify KIF20A-interacting proteins that potentially mediate its effect on gene regulation, we performed V5-tagged KIF20A immunoprecipitation followed by mass spectrometry in MDA-MB-231 cells (Figure 9A). Among the proteins enriched in the KIF20A group, it is interesting that both canonical SWI/SNF complex proteins SMARCA4 and ARID1A were identified as potential KIF20A interaction partners. The SWI/SNF complex is composed of evolutionarily conserved core subunit protein and variant subunit protein. The major subclasses of the human SWI/SNF are BRG1-associated factor (BAF) and polybromo BRG1-associated factor (PBAF). SMARCA4 is one of the evolutionarily conserved core subunits found in BAF and PBAF, while ARID1A is specific to BAF complex (36). Therefore, we decided to characterize the role of these SWI/SNF complex proteins in regulating the effect of KIF20A. We performed coimmunoprecipitation (co-IP) in MDA-MB-231 cells expressing exogenous HA-tagged KIF20A and V5-tagged SMARCA4. We found strong KIF20A-SMARCA4 interactions from either HA- or V5-tagged co-IP (Supplemental Figure 9, A and B). Moreover, their interaction was further validated in co-IP using exogenous V5-tagged KIF20A and endogenous SMARCA4 in both MDA-MB-231 and HCC1806 cells (Figure 9, B and C). Although exogenous KIF20A interacts with V5-tagged ARID1A in MDA-MB-231 and HCC1806 cells (Supplemental Figure 9, C and D), we could not detect its reliable binding to endogenous ARID1A (Supplemental Figure 9, E and F). To support the co-IP results demonstrating the interaction between KIF20A and SMARCA4, we examined their subcellular localization by extracting nuclear and cytoplasmic proteins from MDA-MB-231 and HCC1806 cells. Western blotting results revealed that KIF20A is localized in both the nucleus and cytoplasm, while SMARCA4 is exclusively localized in the nucleus (Figure 9, D and E). Immunofluorescence stains using primary antibodies against endogenous KIF20A and SMARCA4 revealed that both proteins predominantly colocalize in the nucleus (Figure 9F). We also performed immunofluorescence staining in the cells with ectopic overexpression of KIF20A and SMARCA4 and found similar results (Supplemental Figure 9G). Based on these results, we decided to focus on SMARCA4A to elucidate the mechanism of KIF20A in BCSC regulation.

**Figure 10:**
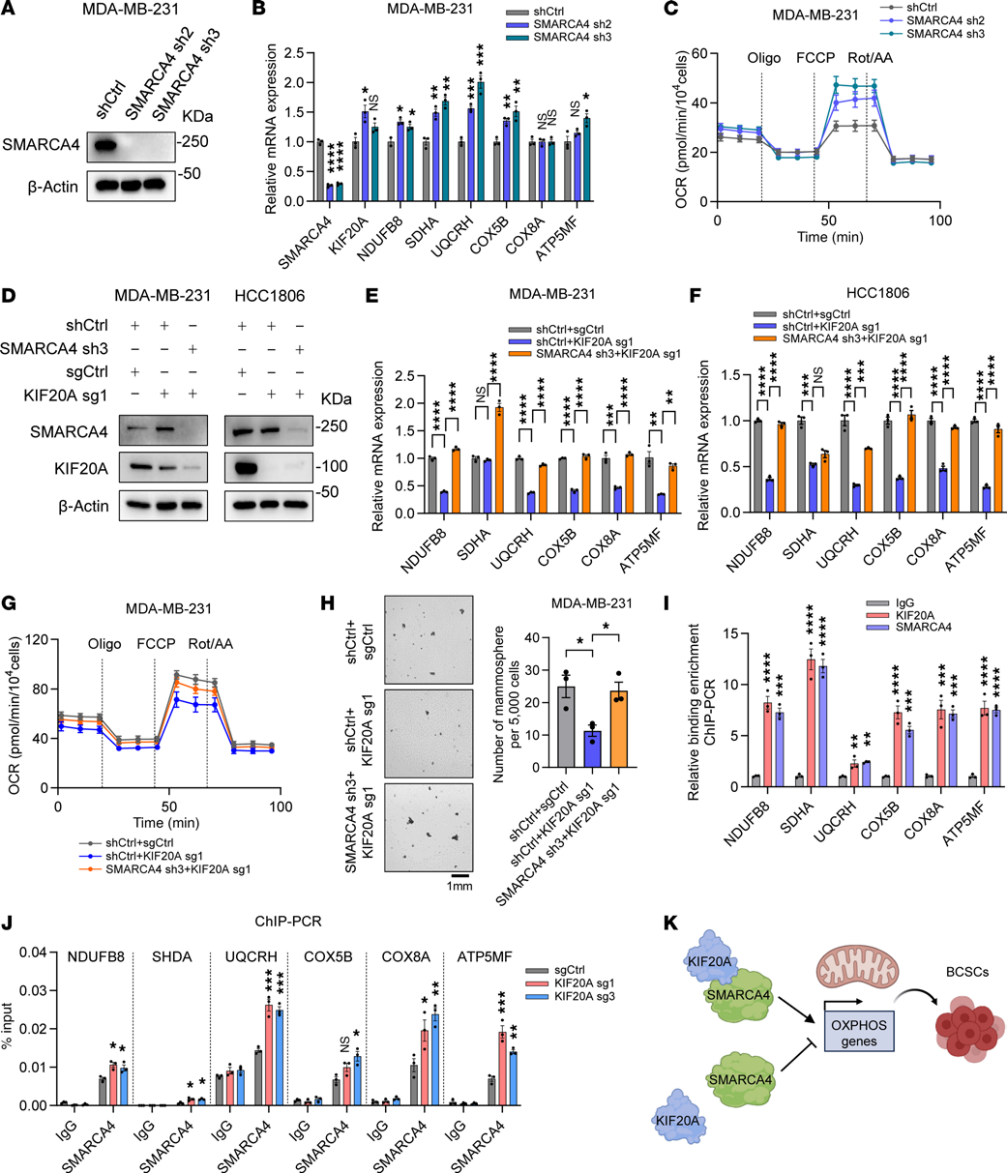
KIF20A controls OXPHOS genes and BCSCs through SMARCA4. (**A**–**C**) Immunoblot analysis of SMARCA4 protein (**A**), RT-qPCR analysis of OXPHOS genes (*n* = 3) (**B**), and measurement of OCR (**C**) in MDA-MB-231 cells transduced with indicated control or SMARCA4 shRNAs. (**D**–**F**) Immunoblot analysis of KIF20A and SMARCA4 (**D**) and RT-qPCR analysis of OXPHOS genes in MDA-MB-231 (**E**) and HCC1806 (**F**) cells transduced with control or KIF20A sgRNA and control or SMARCA4 shRNA as indicated. *n* = 3. (**G** and **H**) Measurement of OCR (*n* = 5) (**G**) and mammosphere formation assay and corresponding quantifications (*n* = 3) (**H**) in MDA-MB-231 cells transduced with control or KIF20A sgRNA and control or SMARCA4 shRNA as indicated. Scale bar: 1 mm. (**I**) ChIP-PCR analysis of KIF20A and SMARCA4 binding at the promoters of OXPHOS genes in MDA-MB-231 cells. *n* = 3. (**J**) ChIP-PCR analysis of SMARCA4 binding at the promoters of OXPHOS genes in MDA-MB-231 cells transduced with control or KIF20A sgRNAs. *n* = 3. (**K**) Schematic model of the mechanism proposed for this study. Each data point represents a biological replicate. Data represent mean ± SEM. Statistical analyses were conducted by 1-way ANOVA with Dunnett’s test (**B**, **I**, and **J**) or with Tukey’s test (**E**, **F**, and **H**). *P < 0.05, **P < 0.01, ***P < 0.001, ****P < 0.0001.

SMARCA4, as part of the SWI/SNF chromatin remodeling complex, regulates transcription through nucleosome repositioning. In mammalian cells, it has been reported that the SWI/SNF complex plays roles in both the activation and repression of transcription (36). We hypothesized that KIF20A interacts with SMARCA4 to influence the transcriptional regulation of BCSCs in TNBC. To first examine the effect of SMARCA4 on OXPHOS gene expression, we depleted SMARCA4 with 2 independent shRNAs in MDA-MB-231 cells (Figure 10A). SMARCA4 knockdown increased OXPHOS gene expression and OCR (Figure 10, B and C), suggesting that SMARCA4 negatively regulates OXPHOS in TNBC. Next, to investigate whether SMARCA4 mediates the expression of OXPHOS genes regulated by KIF20A in TNBC, we further knocked down SMARCA4 in TNBC cells with KIF20A depletion (Figure 10D). Consequently, we found that SMARCA4 knockdown could rescue the reduction of OXPHOS gene expression and OCR in KIF20A-depleted MDA-MB-231 and HCC1806 cells (Figure 10, E–G). In line with OXPHOS gene rescue, knockdown of SMARCA4 restored the decreased mammosphere formation ability in MDA-MB-231 cells caused by KIF20A depletion (Figure 10H).

To determine whether the KIF20A-SMARCA4 complex directly regulates OXPHOS genes, we performed chromatin immunoprecipitation (ChIP) assays in MDA-MB-231 cells using antibodies against KIF20A and SMARCA4, followed by qPCR analysis with primers specific to the promoter regions of OXPHOS genes. We found significant enrichment of OXPHOS gene promoter regions in both KIF20A and SMARCA4 groups compared with the IgG control (Figure 10I). Our ChIP-PCR data for SMARCA4 is consistent with published ChIP sequencing data performed in MDA-MB-231 cells (37) (Supplemental Figure 10). These data suggest that KIF20A and SMARCA4 coordinate the regulation of OXPHOS gene expression by co-occupying their promoters. Given that KIF20A and SMARCA4 play opposing roles in promoting and repressing OXPHOS gene expression, respectively (Figure 6, H and I, and Figure 10B), we hypothesize that KIF20A may antagonize SMARCA4’s regulatory function at OXPHOS genes. To investigate whether KIF20A influences SMARCA4’s promoter-binding activity, we performed SMARCA4 ChIP-PCR analysis in scrambled control sgRNA or KIF20A-knockdown (sg1 and sg3) cells. Notably, we found that KIF20A depletion enhanced SMARCA4 occupancy at the OXPHOS gene promoters (Figure 10J), suggesting that KIF20A antagonizes SMARCA4 binding to relieve repression of OXPHOS genes. Together, these data suggest that KIF20A, by forming a complex with SMARCA4, controls OXPHOS gene expression important in regulating BCSCs in TNBC (Figure 10K).

### KIF20A inhibitor treatment sensitizes TNBC xenografts to chemotherapy.

**Figure 11:**
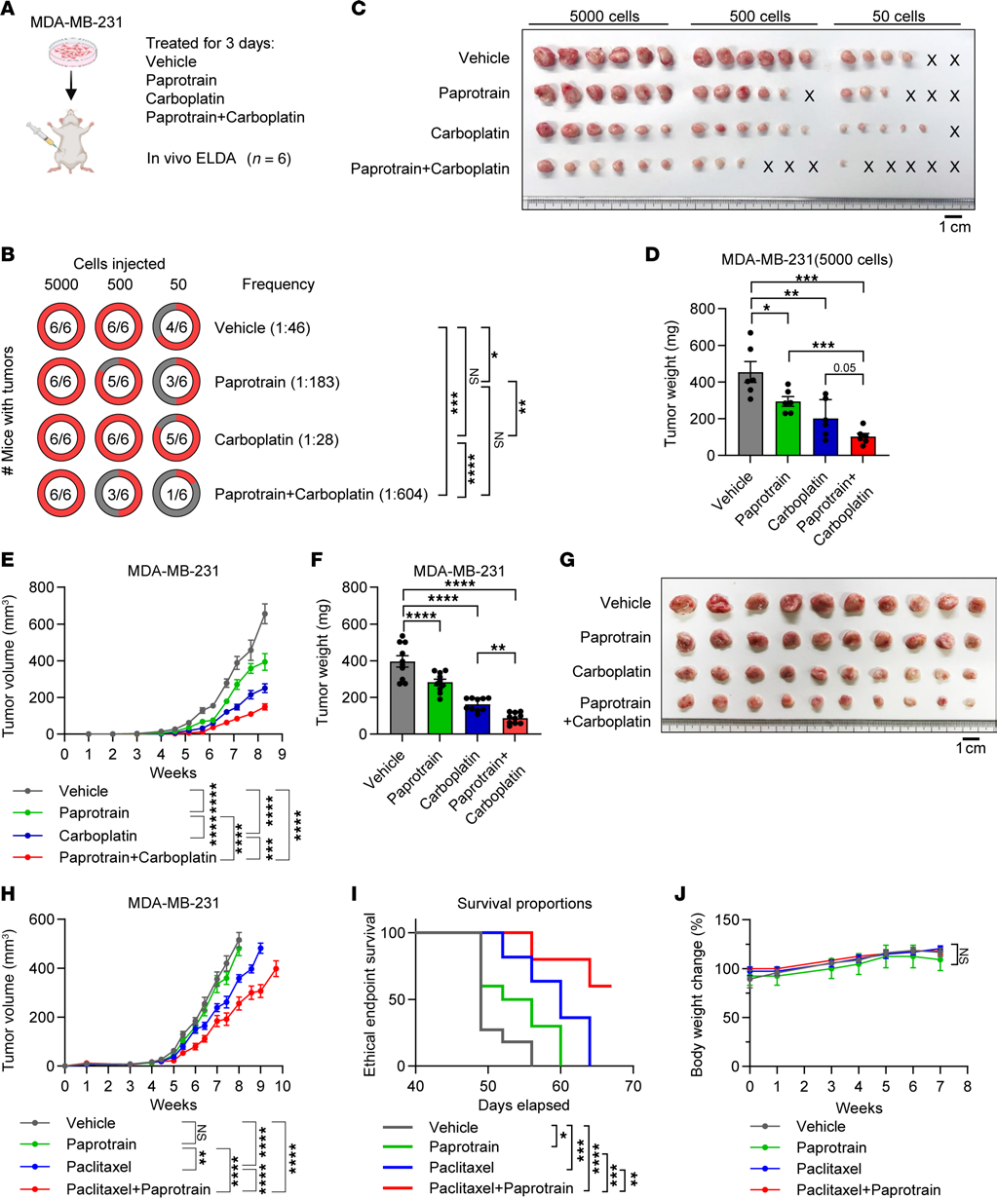
Paprotrain treatment sensitizes TNBC tumor model to chemotherapy. (**A**–**D**) Study design (**A**), limiting dilution tumor-initiating assay (**B**), harvested tumor images (**C**), and tumor weight at the end point with 5,000 cells injected (**D**) for MDA-MB-231 cells treated with vehicle, paprotrain (2 μM), carboplatin (2 μM), or combination. *n* = 6. Scale bar: 1 cm. (**E**–**G**) Tumor growth (**E**), tumor weight upon dissection (**F**), and tumor images (**G**) of MDA-MB-231 orthotopic xenograft model treated with vehicle, paprotrain (50 mg/kg/wk), carboplatin (10 mg/kg/wk), or paprotrain (50 mg/kg/wk) combined with carboplatin (10 mg/kg/wk). *n* = 10. Scale bar: 1 cm. (**H**–**J**) Tumor growth (**H**), mouse survival (**I**), and body weight change (**J**) of MDA-MB-231 orthotopic xenograft model treated with vehicle, paprotrain (50 mg/kg/wk), paclitaxel (10 mg/kg/wk), or paprotrain (50 mg/kg/wk) combined with paclitaxel (10 mg/kg/wk). *n* = 10–11. Data represent mean ± SEM. Statistical analyses were conducted by χ^2^ test (**B**), 1-way ANOVA with Tukey’s test (**D** and **F**), 2-way ANOVA with Tukey’s test (**E**, **H**, and **J**), or log-rank (Mantel-Cox) test (**I**). *P < 0.05, **P < 0.01, ***P < 0.001, ****P < 0.0001.

Platinum-based chemotherapy, such as carboplatin, is frequently used in the treatment of TNBC (38). However, acquired resistance to prolonged platinum-based chemotherapy has been reported, often due to BCSCs (39). Given that the KIF20A inhibitor effectively targets BCSCs (Figure 5), while conventional chemotherapy such as carboplatin efficiently debulks the bulk tumor cells, we evaluated the therapeutic efficacy of combining carboplatin with paprotrain in vivo. To determine whether paprotrain and carboplatin display a combinatorial effect on BCSCs, we pretreated MDA-MB-231 tumor cells with carboplatin, paprotrain, or their combination, alongside vehicle-treated controls. Treated cells were then subjected to secondary in vivo extreme limiting dilution assays (ELDAs) to assess BCSC frequency (Figure 11A). We found that paprotrain alone reduced the tumor initiation rate, whereas carboplatin treatment had minimal effect, consistent with previous findings (40, 41). Notably, the combination treatment further suppressed tumor initiation and tumor burden compared with all other treatment groups (Figure 11, B–D). Motivated by these findings, we treated MDA-MB-231 tumor-bearing mice with the same regimen and observed that the combination of carboplatin and paprotrain further reduced tumor growth and weight compared with carboplatin alone (Figure 11, E–G).

It has been reported that BCSCs are associated with resistance to paclitaxel, another widely used chemotherapeutic agent (13). Notably, a study has shown that KIF20A expression is downregulated in paclitaxel-sensitive breast cancer cells but elevated in paclitaxel-resistant ones (42). Therefore, we also examined the therapeutic efficacy of combined treatment with paclitaxel (10 mg/kg/wk) and paprotrain (50 mg/kg/wk) in an MDA-MB-231 orthotopic xenograft model. As a result, combined therapy significantly reduced tumor growth and increased mouse survival compared with treatment with paclitaxel or paprotrain alone (Figure 11, H and I). Importantly, no significant change in body weight was observed in combined therapy, suggesting that it is a well-tolerated treatment (Figure 11J). Taken together, our results suggest that KIF20A inhibition with specific inhibitors, such as paprotrain, could sensitize TNBC to standard chemotherapy, potentially by reducing BCSC fitness in vivo.

## Discussion

In this study, we identified KIF20A as a regulator in BCSCs of TNBC. In summary, transcriptomic profiling of BCSCs versus non-BCSCs revealed KIF20A to be essential for sustaining abilities of self-renewal, population expansion, and tumor initiation of BCSCs. Interestingly, in vitro and in vivo experiments using KIF20A-overexpressing cell lines revealed that KIF20A is not sufficient to induce BCSC abilities. KIF20A loss- and gain-of-function studies coupled with transcriptomic and proteomic analyses revealed its regulation of mitochondrial function through OXPHOS genes by forming a complex with the SWI/SNF complex protein SMARCA4. We further demonstrated that OXPHOS sustained BCSC self-renewal ability in vivo. Therapeutically, KIF20A inhibition with paprotrain suppressed BCSC self-renewal, tumor initiation, and tumor growth in TNBC, as well as sensitized TNBC to standard chemotherapy. Therefore, our study identified KIF20A as a novel therapeutic target for BCSCs in TNBC, offering a potential avenue for future therapeutic development.

KIF20A (also known as MKLP2) belongs to the kinesin-6 family, known for its role in intracellular trafficking and mitotic spindle organization during cell division (21, 43). KIF20A expression is elevated in various cancers, including breast cancer (20). Similarly to our findings, suppression of endogenous KIF20A inhibited the growth of cells in breast cancer (20). Furthermore, KIF20A overexpression correlates with poor prognosis and serves as an independent prognosis factor in breast cancer patients (20, 44). Notably, our study presents evidence implicating KIF20A in the regulation of BCSC abilities.

Several studies have investigated the role of KIF20A in cancer. KIF20A has been shown to arrest breast cancer cell division at G_2_/M phase, ultimately leading to cell death (44). Additionally, in glioma, KIF20A depletion disrupts cell division processes, leading to decreased cell proliferation and increased apoptosis (45). In another study, KIF20A colocalized with discs large MAGUK scaffold protein 5 (DLG5) in pancreatic cancer, a protein known to interact with cell cycle regulators and implicated in tumorigenesis (46). While these studies link KIF20A to cell cycle and cell division control, our study explores its connection to metabolism, specifically OXPHOS, in breast cancer.

BCSCs exhibit a distinct metabolic profile, reportedly switching between glycolysis and OXPHOS to adapt to various environments (31, 33). While the debate regarding the primary metabolic pathway in cancer stem cells continues, our findings indicated that OXPHOS plays a crucial role in maintaining the abilities of BCSCs, such as self-renewal. Supporting this notion, a previous study demonstrated that metformin, an OXPHOS inhibitor, reduced mammosphere formation and *OCT4* expression, a marker of BCSCs, in breast cancer (47). These findings align with and strengthen the conclusion drawn from our own research.

Furthermore, accumulating evidence suggests a link between metabolic plasticity toward OXPHOS in BCSCs and therapy resistance. Specifically, in TNBC, chemotherapy can induce a switch toward OXPHOS, leading to elevated ROS. This increase in ROS promotes the stabilization of hypoxia-inducible factor 1α (HIF-1α), ultimately contributing to chemoresistance in BCSCs (32). Additionally, studies have shown that BCSCs in TNBC resistant to mTORC1/2 inhibitors exhibit activation of Notch signaling, which appears to be dependent on OXPHOS (48). These findings collectively highlight the potential of targeting OXPHOS as a therapeutic strategy to improve the prognosis of TNBC by reducing the ability of BCSCs.

Our study reveals that KIF20A collaborates with SMARCA4 in regulating OXPHOS genes in BCSCs. SMARCA4 is a component of the SWI/SNF chromatin remodeling complex, known to modulate gene expression through nucleosomes (36). Previous studies have shown that SMARCA4 mutations in lung cancer cells enhance OXPHOS activity and ROS production. Notably, those studies also showed that SMARCA4 mediated expression of energy stress response genes such as *HIF2A* and hexokinase 2 (*HK2*) (49). Interestingly, the same study found that the OXPHOS inhibitor suppressed cancer cell proliferation when SMARCA4 loss triggered a shift from glycolysis to OXPHOS (49). One limitation of our current study is that KIF20A and SMARCA4 likely regulate a broad set of genes that may be critical for BCSC function but were not examined here, as suggested by our RNA-Seq analysis and the partial rescue phenotype observed with mt-ND1. In addition, although we observed a substantial nuclear localization of KIF20A, it remains unclear whether this subcellular distribution is critical for maintaining BCSC stemness or specifically involved in modulating SMARCA4 function. Therefore, our finding that KIF20A represses SMARCA4-mediated transcription warrants further investigation to elucidate the distinct roles of KIF20A and SMARCA4 in regulating OXPHOS or other transcriptional programs in BCSCs. Overall, our results identified KIF20A as a novel therapeutic target for BCSCs in TNBC. By regulating BCSC plasticity, KIF20A offers a promising avenue for improving treatment efficacy.

## Methods

### Sex as a biological variable.

Our study exclusively examined female mice because the disease modeled is only relevant in females.

### Cell culture.

Hs578T, MDA-MB-231, and 293T cells were cultured in DMEM (Gibco, 11965118) supplemented with 10% fetal bovine serum (FBS) and 1% penicillin-streptomycin. MDA-MB-468, HCC3153, HCC70, HCC1143, HCC1187, and HCC1806 cells were cultured in RPMI 1640 (Gibco, 11875093) supplemented with 10% FBS and 1% penicillin-streptomycin. SUM149 cells were cultured in HuMEC Ready Medium (Gibco, 12752010). HCC3153 cells were obtained from the cell repository of the Hamon Center for Therapeutic Oncology Research, UT Southwestern Medical Center. All other cell lines were obtained from ATCC. The cells were incubated at 37°C in a humidified atmosphere containing 5% CO_2_. Mycoplasma testing was routinely carried out with MycoAlert PLUS Mycoplasma Detection Kit (Lonza, LT07-703) to ensure cells were mycoplasma free.

### Flow cytometry and FACS.

Cultured tumor cells were digested with trypsin-EDTA (Thermo Fisher Scientific, 25200056), dissociated into single cells, centrifuged, washed, and resuspended in PBS containing 2% FBS. Cell numbers were determined using a TC20 Automated Cell Counter (Bio-Rad, 1450102).

For CD24/CD44 staining, 5 × 10^5^ cells were incubated on ice for 30 minutes with APC–anti-CD24 (BioLegend, 311118) and PerCP-Cy5.5–anti-CD44 (BioLegend, 338820). After washing twice with 2% FBS/PBS, cells were resuspended in PBS containing 2% FBS and DAPI (300 nM). For quantification of ALDH activity via the ALDEFLUOR assay (STEMCELL Technologies, 01700), cellular suspensions containing 1 × 10^6^ viable cells per sample were processed following standardized protocols. Each biological specimen required parallel preparation of experimental and negative control aliquots. Precisely 1 mL of cell suspension in ALDEFLUOR Assay Buffer was aliquoted into designated test tubes. The control tubes received 5 μL of diethylaminobenzaldehyde (DEAB) inhibitor solution (ALDEFLUOR DEAB Reagent) with immediate sealing of both tube and reagent stock. Experimental aliquots were supplemented with 5 μL of activated BODIPY-aminoacetaldehyde substrate (BAAA, ALDEFLUOR Reagent). After vortexing, 500 μL of the reaction mixture was rapidly transferred to DEAB-containing controls to establish inhibition baselines. Both experimental and control groups underwent synchronized incubation at 37°C for 30–60 minutes, strictly maintaining temporal parameters below 60-minute threshold. Post-incubation centrifugation at 130 *g* for 5 minutes facilitated supernatant removal. Cellular pellets were resuspended in 500 μL ice-cold assay buffer and kept on ice.

Fluorescence signals were analyzed using BD FACSCanto II, and cell sorting was performed using BD FACSAria III (BD Biosciences). Spectral compensation and gating optimization used unstained controls and single-channel reference samples.

### RNA sequencing (RNA-Seq).

Total RNA was extracted from tumor cell lysates (biological triplicates per group) using the RNeasy Mini Kit (QIAGEN, 74104) with on-column DNase I treatment to remove genomic DNA. RNA integrity was verified to meet quality standards for sequencing. Stranded mRNA libraries were prepared using the TruSeq Stranded mRNA Library Prep Kit (Illumina) via poly-A selection, fragmentation, cDNA synthesis, end repair, adapter ligation, and PCR amplification. Paired-end sequencing was performed on an Illumina NovaSeq platform (Novogene Co. Ltd.). Processed reads were aligned to the human reference genome (GRCh38), and differential expression analysis was performed using DESeq2 (v1.38.3) with independent filtering. Differentially expressed genes (DEGs) were defined as those with |log_2_(fold change)| > threshold and *P* < 0.05. Gene set enrichment analysis (GSEA) was carried out using the GSEA software with Hallmark gene sets. *Z* scores were computed per gene by subtracting the mean and dividing by the standard deviation. Heatmaps and enrichment plots were generated using GraphPad Prism.

### Lentivirus packaging and infection.

Lentiviral particles were produced in 293FT cells (Thermo Fisher Scientific, R70007) by cotransfection with lentiviral expression plasmids together with pSPAX2 (Addgene, 12260) and pMD2.G (Addgene, 12259) using Lipofectamine 3000 and P3000 reagent (Thermo Fisher Scientific, L3000015) following the manufacturer’s instructions. Viral supernatants were collected at 48 and 72 hours after transfection, pooled, filtered through a 0.45 μm filter (MilliporeSigma, SLHV033RS), and stored at –80°C.

For infection, target cells were plated 1 day earlier to reach approximately 30% confluence and then incubated with viral supernatant supplemented with 8 μg/mL Polybrene (MilliporeSigma, TR-1003-G) for 24 hours. After infection, culture medium was replaced with fresh growth medium containing puromycin (2 μg/mL; Thermo Fisher Scientific, A1113803) for 2–3 days. Selection efficiency was confirmed by complete cell death in noninfected control wells.

### Western blotting.

Cells were collected and lysed in either RIPA lysis buffer (50 mM Tris-HCl [pH 8.0], 150 mM NaCl, 1% NP-40, 0.5% sodium deoxycholate, 0.1% SDS) or EBC buffer (50 mM Tris-HCl [pH 8.0], 120 mM NaCl, 0.5% NP-40) supplemented with protease inhibitor cocktail (cOmplete, Roche, 04693132001). Lysates were incubated on ice for 30 minutes and cleared by centrifugation (12,000*g*, 15 minutes, 4°C). Protein concentrations were determined using the Pierce Bradford Protein Assay Kit (Thermo Fisher Scientific, 23200) with bovine serum albumin (BSA) as the standard. Equal amounts of protein (20–50 μg) were mixed with loading buffer containing 2% SDS and 5% β-mercaptoethanol, denatured at 100°C for 10 minutes, and separated by SDS-PAGE. Proteins were transferred to nitrocellulose membranes (Thermo Fisher Scientific, 88018) and blocked in 5% nonfat milk in TBST. Membranes were incubated with primary antibodies overnight at 4°C, followed by HRP-conjugated secondary antibodies (1:5,000; Cell Signaling Technology, 31430 or 31460) for 1 hour at room temperature. Signals were visualized using enhanced chemiluminescence (ECL; Millipore, WBULS0100) and imaged with a ChemiDoc Imaging System (Bio-Rad) using Image Lab software.

Primary antibodies used were as follows: KIF20A (1:1,000; Proteintech, 15911-1-AP), SMARCA4/BRG1 (1:1,000; Cell Signaling Technology, 49360S), histone H3 (1:2,000; Cell Signaling Technology, 4499T), ARID1A (1:100; Santa Cruz Biotechnology, SC32761), MVB12B (1:200; Sigma-Aldrich, HPA043683), V5 tag (1:1,000; Cell Signaling Technology, 13202S), HA tag (1:1,000; Cell Signaling Technology, 3724S), FLAG tag (1:1,000; Cell Signaling Technology, 14793S), vinculin (1:5,000; Sigma-Aldrich, V9131), α-tubulin (1:1,000; Cell Signaling Technology, 3873S), and β-actin (1:1,000; Cell Signaling Technology, 3700S).

### Quantitative reverse transcription PCR.

Total RNA was extracted with RNeasy Mini Kit (QIAGEN, 74104) following the manufacturer’s protocol. RNA purity and concentration were quantified via UV spectrophotometry (Thermo Fisher Scientific, NanoDrop). cDNA was synthesized using an iScript cDNA Synthesis Kit (Bio-Rad, 1708890). qPCR reactions were performed in triplicate using the iTaq Universal SYBR Green Supermix (Bio-Rad, 1725124) on a CFX384 Touch Real-Time PCR Detection System (Bio-Rad). Target gene expression was normalized to endogenous reference genes (GAPDH/β-actin). The relative expression was calculated using the 2^−ΔΔCt^ method. qPCR primers are listed in Supplemental Table 3.

### Mammosphere formation assay.

Human breast carcinoma cells were enzymatically dissociated into single-cell suspensions using trypsin-EDTA (Thermo Fisher Scientific, 25200056). Viable cells (≥95% viability via trypan blue exclusion) were resuspended in MammoCult Human Medium Kit (STEMCELL Technologies, 05620) supplemented with the following additives: recombinant heparin sodium (4 μg/mL; STEMCELL, 07980), hydrocortisone (1 μg/mL; Sigma-Aldrich, H0888), penicillin-streptomycin (1% vol/vol; Gibco 15140-122). Cell suspensions were seeded at clonal density (2.5 × 10^3^ cells/mL) into 6-well ultra-low-attachment plates (Corning Costar, 3471) to prevent extracellular matrix adhesion. Cultures were maintained under conditions of 5% CO_2_, 37°C, with strict humidity control for 7–14 days. Fresh medium was added every 72 hours using pre-equilibrated MammoCult complete medium to maintain nutrient/gas homeostasis while minimizing mechanical perturbation of developing mammospheres. Primary mammospheres (diameter ≥50 μm) were quantified at approximately day 10 using phase-contrast microscopy. Digital image acquisition was performed with microscopy, followed by sphere counting via ImageJ (NIH) using optimized threshold parameters. Sphere-forming efficiency was calculated as: (number of mammospheres / initial seeded cells) × 100%.

### Extreme limiting dilution assay.

Extreme limiting dilution assay (ELDA) was implemented to mathematically determine the frequency of breast cancer stem cells (BCSCs) exhibiting mammosphere-initiating capacity. Single-cell suspensions derived from tumor cell culture were prepared using enzymatic dissociation (trypsin-EDTA, Thermo Fisher Scientific, 25200056) coupled with mechanical trituration through 27G needles. Cell viability was confirmed to be greater than 95% via trypan blue exclusion. Serially diluted cell suspensions were plated in 96-well ultra-low-attachment microplates (Corning Costar, 3474). Experimental cohorts included logarithmic dilutions: 5, 50, 500, 5,000 cells per well, suspended in MammoCult complete medium (STEMCELL Technologies, 05620) supplemented with recombinant heparin sodium (4 μg/mL; STEMCELL Technologies, 07980), hydrocortisone (1 μg/mL; Sigma-Aldrich, H0888), penicillin-streptomycin (1% vol/vol; Gibco, 15140-122). Plates were maintained under conditions of 5% CO_2_, 37°C. Fresh medium was added every 72 hours to preserve developing mammospheres. Mammosphere-positive wells were identified using bright-field imaging with a ×4 objective. Spheres were counted and frequency and statistical significance of mammosphere-initiating cells (BCSCs) were calculated using the ELDA software (http://bioinf.wehi.edu.au/software/elda/) (24). Stem cell frequency was expressed as 1/BCSC frequency with confidence limits.

### Limiting dilution tumor-initiating assay and breast tumorigenicity in mice.

Human breast tumor cells were enzymatically dissociated into single-cell suspensions using pre-warmed trypsin-EDTA (Thermo Fisher Scientific, 25200056) at 37°C, followed by mechanical trituration through 40 μm cell strainers (Falcon, 352340). Viable cells (≥95% viability, assessed by trypan blue exclusion) were counted with a TC20 Automated Cell Counter (Bio-Rad, 1450102) and adjusted to 1 × 10^4^ to 1 × 10^7^ cells/mL in ice-cold FBS (Gibco, 10437-028). Cell suspensions were mixed 1:1 (vol/vol) with growth factor–reduced Matrigel Matrix (Corning, 356234) under sterile conditions at 0°C–4°C to prevent premature polymerization.

Six-week-old female NOD.Cg-*Prkdc^scid^Il2rg^tm1Wjl^*/SzJ (NSG) mice (The Jackson Laboratory, 005557) were acclimatized for 7 days in specific pathogen–free facilities. Before implantation, mice were anesthetized with isoflurane and positioned in lateral recumbency. A 100 μL suspension containing tumor cells (dose range: 5 × 10^2^ to 5 × 10^5^ cells) in Matrigel/FBS mixture was injected into the fourth mammary fat pad using a 27G insulin syringe. Palpation-based tumor detection commenced on day 7 after inoculation. Bidimensional measurements (major/minor axis) were obtained weekly using digital calipers. Tumor volume was calculated via modified ellipsoid formula: volume (mm^3^) = (length × width^2^) × 0.5. Humane endpoints were strictly enforced: any tumor exceeding 1,500 mm^3^ (equivalent to ~1.5 cm diameter sphere) or exhibiting ulceration/necrosis triggered immediate cohort euthanasia via CO_2_ asphyxiation followed by cervical dislocation. Terminal tumors were excised, weighed, and divided for parallel analyses.

To perform limiting dilution tumor-initiating assay, tumorigenicity in mice was carried out as described above in a series of groups with descending numbers of cells injected into mammary gland, e.g., 5,000, 1,000, 500, 200, 40 cells or 5,000, 500, 50 cells per group. After inoculation, tumor initiation was observed weekly. About 2–4 months later, CSC frequency and statistical significance were calculated according to tumor incidence in each group using the ELDA software (http://bioinf.wehi.edu.au/software/elda/) (24). Our study exclusively examined female mice because the disease modeled is only relevant in females. All animal experiments were conducted in accordance with NIH guidelines and were approved by the Institutional Animal Care and Use Committee (IACUC) of University of Texas Southwestern Medical Center (protocol 2019-102794).

### Immunofluorescence staining.

Cells were fixed using 4% formaldehyde for 15 minutes at room temperature. Unspecific binding of the antibodies was blocked with BSA for 1 hour. The cells were incubated with 1:1,000 dilution of KIF20A primary antibody (Proteintech, 15911-1-AP) or 1:800 dilution of SMARCA4 primary antibody (Proteintech, 66561-1-Ig) at 4°C overnight followed by 1:1,000 dilution of secondary antibody [Donkey anti-Rabbit IgG ReadyProbes, Alexa Fluor 488, Invitrogen, R37118; and Goat anti-Mouse IgG (H+L) Cross-Adsorbed Secondary Antibody, Alexa Fluor 647, Invitrogen, A-21235]. DNA staining was performed using 1:5,000 dilution of DAPI (Sigma-Aldrich, D9542-10MG). Images were obtained using a Resolve fluorescence microscope (Discover Echo) or confocal microscope.

### OCR measurement.

OCR measurement was conducted using the Seahorse XFe24 Analyzer (Agilent Technologies). About 5 × 10^4^ to 8 × 10^4^ cells were seeded into the Seahorse XF24 Cell Culture Microplate the day before assay, and cells were cultured overnight with the appropriate cell culture growth medium. Meanwhile, a sensor cartridge of the Seahorse XFe24 Extracellular Flux Assay Kit was hydrated with the XF Calibrant at 37°C in a non-CO_2_ incubator overnight. The next day, the medium was changed with freshly prepared and pre-warmed Seahorse FX DMEM medium (Agilent Technologies, 103575-100) supplemented with 1 mM pyruvate, 2 mM glutamine, and 10 mM glucose, and then cells were incubated at 37°C in a non-CO_2_ incubator for 45 minutes to 1 hour. The inhibitors from the Seahorse XF Cell Mito Stress Test Kit (Agilent Technologies, 103015-100) — oligomycin, carbonyl cyanide 4-trifluoromethoxyphenylhydrazone (FCCP), and a mix of rotenone and antimycin (Rot/AA) — were loaded into ports of a sensor cartridge so that the final concentration at the time of measurement was 2.0 μM, 2.0 μM, and 0.5 μM, respectively. The plates were analyzed by the Seahorse machine according to the manufacturer’s instructions. Data were normalized against cell number.

### Tandem affinity purification tagging and mass spectrometry analysis.

Cells were harvested in lysis buffer (50 mM Tris-HCl [pH 7.5], 150 mM NaCl, 0.5% NP-40, 10% glycerol) supplemented with protease and phosphatase inhibitors and adjusted to a final protein concentration of 10–20 μg/μL. After centrifugation, equal amounts of lysates were incubated with anti-V5 beads (Sigma-Aldrich) overnight at 4°C with rotation. Beads were washed 5 times with lysis buffer, and proteins were eluted by boiling in SDS-PAGE sample buffer. Samples were resolved about 2 cm on SDS-PAGE, and gel pieces were excised, reduced, alkylated, and subjected to tryptic digestion. Peptides were extracted and desalted using homemade C18 stage tips.

For mass spectrometry, peptides were dissolved in 0.1% formic acid and analyzed on a Q-Exactive HF-X coupled to an Easy-nLC 1200 (Thermo Fisher Scientific). Peptides were loaded onto a nanoEase MZ HSS T3 Column (100 Å, 1.8 μm, 75 μm × 250 mm; Waters) and separated with a 50-minute gradient at 250 nL/min: 5%–30% buffer B over 29 minutes, 30%–45% buffer B over 6 minutes, ramp to 100% buffer B in 1 minute, followed by a 14-minute wash (buffer A: 0.1% formic acid; buffer B: 80% acetonitrile, 0.1% formic acid). Liquid chromatography–mass spectrometry was performed in data-dependent mode: full mass spectrometry at 60,000 resolution (*m*/*z* 200, <5 ppm) followed by higher-energy collisional dissociation–tandem mass spectrometry of the top 15 ions at 15,000 resolution, normalized collision energy 27 eV, and dynamic exclusion of 20 seconds. Three technical replicates were acquired per sample.

Raw data were processed using MaxQuant v1.6.10.43 against the UniProt human database (UP000005640). FDR for peptide-spectrum matches and protein identifications was set to 1%. Search parameters allowed up to 2 missed cleavages, oxidation of methionine, and protein N-terminal acetylation as variable modifications, with carbamidomethylation of cysteine as a fixed modification. Peptide identifications were filtered to exclude reverse and contaminant entries. Label-free quantitation was performed in MaxQuant (https://www.maxquant.org). Statistical analysis, including 2-sample *t* tests (*P* < 0.05), was conducted in Perseus v1.6.10.50 to identify significant changes in protein abundance.

### Coimmunoprecipitation.

Whole-cell lysates were prepared using RIPA buffer (50 mM Tris-HCl [pH 8.0], 150 mM NaCl, 1% NP-40, 1 mM EDTA, 0.5% sodium deoxycholate, 0.1% SDS) supplemented with protease and phosphatase inhibitors at 4°C. Lysates were clarified by centrifugation and incubated overnight at 4°C. Subsequently, lysates were incubated with HA- or V5-conjugated beads (Roche, 11815016001; MilliporeSigma, A7345) for 4 hours at 4°C. Beads were washed 3 times with RIPA buffer, and bound proteins were eluted by boiling in SDS loading buffer. Eluates were analyzed by SDS-PAGE followed by Western blotting.

### Subcellular fractionation.

Nuclear and cytoplasmic fractions were obtained using the Nuclear Extraction Kit (Abcam, ab113474) following the manufacturer’s instructions. Adherent cells were washed twice with PBS, detached with trypsin-EDTA, collected, and counted. Cell pellets were resuspended in 1× Pre-Extraction Buffer (Abcam, ab113474), incubated on ice for 10 minutes, vortexed, and centrifuged to collect the cytoplasmic fraction. For nuclear extracts, DTT and protease inhibitor cocktail were added to the extraction buffer, which was applied to the nuclear pellet. Samples were incubated on ice for 15 minutes with vortexing every 3 minutes, then centrifuged at 4°C to collect the nuclear extract.

### ChIP-PCR.

ChIP was performed using the SimpleChIP enzymatic chromatin IP kit (Cell Signaling Technology, 9005) according to the manufacturer’s instructions. MDA-MB-231 cells were subjected to ChIP with anti-KIF20A (Proteintech, 15911-1-AP) and anti-SMARCA4 (Cell Signaling Technology, 49360S) antibodies. Briefly, samples were cross-linked with 1% formaldehyde on a shaker for 10 minutes, followed by the addition of 125 mM glycine to quench unreacted formaldehyde. The samples were then collected by centrifugation at 1,500*g* for 10 minutes at 4°C, followed by cell lysis and sonication. Meanwhile, beads were incubated with anti-KIF20A or anti-SMARCA4 antibodies at 4°C, rotating overnight. The sonicated samples were incubated with the antibody-bound beads overnight at 4°C. The beads were washed 8 times using a magnetic stand, and samples were collected by centrifugation. The protein-DNA complexes were eluted for 30 minutes at 65°C, followed by reversal of cross-links overnight at 65°C. RNA was digested with RNase at 37°C for 1 hour, and proteins were digested with proteinase K at 55°C for 2 hours. DNA was purified using the QIAGEN PCR Purification Kit. The DNA was then analyzed by qPCR using the primers listed in Supplemental Table 3.

### Cell proliferation assay.

For cell proliferation assays, cells were seeded (200 cells per well) in 96-well plates in DMEM and changed with fresh medium every 2 days. At indicated time points, the cells were cultured in 100 μL fresh growth medium supplemented with 10 μL MTS reagents (Abcam, ab197010), followed by incubation at 37°C for 2 hours. OD absorbance values were measured at 490 nm using a BioTek plate reader (Agilent Technologies). For 2D assay, cells were seeded in 6-well plates (1,000 cells per well); after the cells reached a specific density visually, cells were fixed by 5% methanol for 20 minutes and stained with 0.5% crystal violet for 20 minutes. The stained colony was washed with PBS, followed by imaging.

### Statistics.

Statistical analyses were performed using Prism 9.0 (GraphPad Software). Statistical significance between 2 groups was assessed using an unpaired, 2-tailed Student’s *t* test. For data from 3 or more groups, 1- or 2-way ANOVA was conducted, followed by Dunnett’s test or Tukey’s test for *P* value correction in multiple comparisons. For multiple comparisons of survival data, the log-rank (Mantel-Cox) test was applied, followed by Bonferroni’s adjustment of *P* values. ELDA software was used to calculate BCSC frequency and statistical significance. *P* values of less than 0.05 were considered significant. All graphs depict mean ± SEM.

### Study approval.

All procedures on mice were approved by the IACUC of University of Texas Southwestern Medical Center (approval 2019-102794).

### Data availability.

The mass spectrometry proteomics data were deposited to the ProteomeXchange Consortium via the PRIDE (50) partner repository with the dataset identifier PXD060031. RNA-Seq data were deposited in the NCBI’s Gene Expression Omnibus database (GEO GSE298177). Values for all data points are available in the Supporting Data Values file. All other data and reagents relevant to the current study are available on reasonable request excluding confidential patient identity information.

## Author contributions

YA, WC, CL, and QZ conceived and designed the study. YA, WC, RS, L Xu, XC, QZ, NG, and SL acquired data. YA, WC, CL, RS, LG, L Xu, L Xie, XC, QZ, NG, and SL analyzed and interpreted data. YA, WC, CL, RS, HF, MT, QL, FZ, TW, HL, JF, JZ, CZ, HY, LH, LG, L Xu, L Xie, XC, and QZ wrote, reviewed, and/or revised the manuscript. YA, WC, CL, RS, L Xie, XC, and QZ provided administrative, technical, or material support. QZ and CL supervised the study.

## Funding support

This work is the result of NIH funding, in whole or in part, and is subject to the NIH Public Access Policy. Through acceptance of this federal funding, the NIH has been given a right to make the work publicly available in PubMed Central.

- NIH grants R21AG071229, 1R41DK133051-01A1, and R01GM133107-01 (to XC).
- Cancer Prevention and Research Institute of Texas award RR190058 (to QZ).
- 2024 Urology Care Foundation Research Scholar Award Program (to CL).
- Society of Urologic Oncology Specialized Programs of Research Excellence Award (to CL).

## Supplementary Material

Supplemental data

Unedited blot and gel images

Supplemental table 1

Supplemental table 2

Supplemental table 3

Supporting data values

## Version 1 12/15/2025

Electronic publication
