# Supplementary material for: Targeting kinesin family member 20A sensitizes stem-like triple-negative breast cancer cells to standard chemotherapy
Source: J Clin Invest. 2025 Dec 15;135(24):e182394. doi: 10.1172/JCI182394 (PMC12700555; doi:10.1172/JCI182394)
Supplement: Supplemental data [file jci-135-182394-s098.pdf]

Supplemental Data

Targeting kinesin family member 20A sensitizes stem-like triple-negative breast cancer cells to standard chemotherapy

Yayoi Adachi[1,2,#], Weilong Chen[1,3,#], Cheng Zhang[1], Nina Gildor[1], Rachel Shi[1], Haoyong Fu[1], Masashi Takeda[1], Qian Liang[1], Fangzhou Zhao[1], Tao Wang[1], Hongyi Liu[1], Jun Fang[1], Jin Zhou[1], , Hongwei Yao[1], Lianxin Hu[1], Shina Li[1], Lei Guo[4], Lin Xu[4], Ling Xie[5], Xian Chen[5], Chengheng Liao[1,*], Qing Zhang[1,6,*]

[1]Department of Pathology, University of Texas Southwestern Medical Center, Dallas, TX 75390, USA
[2]Department of Breast and Endocrine Surgery, Nagoya University Graduate School of Medicine, Nagoya, Aichi, 4668560, Japan
[3]Jinfeng Laboratory, Chongqing 400039, P. R. China
[4]Quantitative Biomedical Research Center, Peter O'Donnell Jr. School of Public Health, University of Texas Southwestern Medical Center, Dallas, TX 75390, USA
[5]Department of Biochemistry and Biophysics, University of North Carolina, Chapel Hill, NC 27599, USA
[6]Simmons Comprehensive Cancer Center, University of Texas Southwestern Medical Center, Dallas, Texas, USA
[#] Contributed equally.

Supplemental Figure 1-10

A

Cell line	CD24⁻CD44⁺ %	ALDH⁺ %
MDA-MB-231	99.0	3.87
MDA-MB-468	9.27	4.30
SUM149	13.8	32.0
Hs578T	89.7	16.4
HCC1143	0.044	31.2
HCC1187	0.30	70.4
HCC3153	1.98	7.53
HCC70	17.5	38.3
HCC1806	17.8	91.9

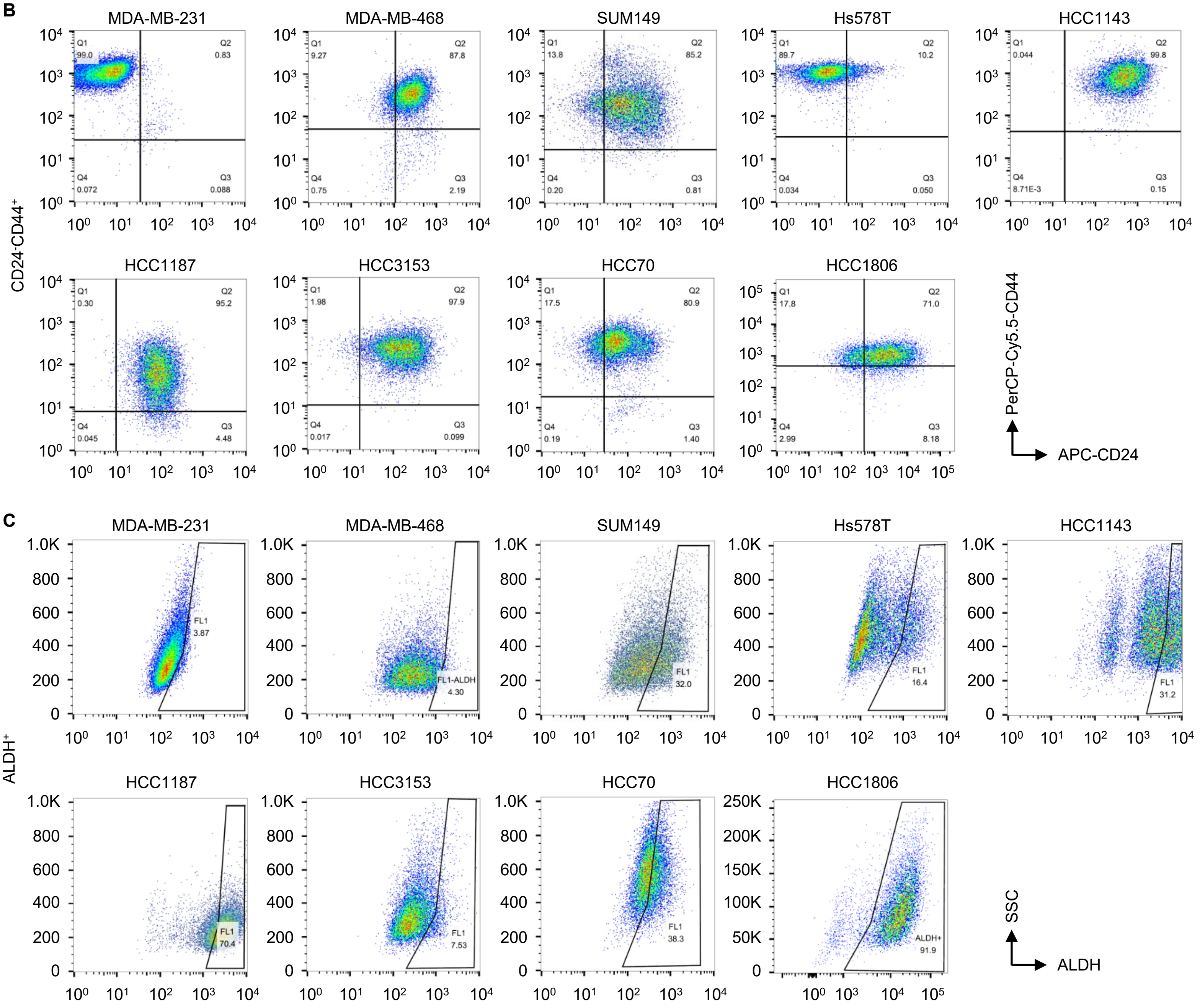

Supplemental Figure 1. Characterization of the BCSC populations in various TNBC cells. (**A**) Summary of the percentage of CD24⁻CD44⁺ or ALDH+ populations quantified by flow cytometry in indicated TNBC cells. (**B**) Flow cytometry plots showing CD24⁻CD44⁺ populations. (**C**) Quantification of ALDH⁺ cells using the ALDEFLUOR assay.

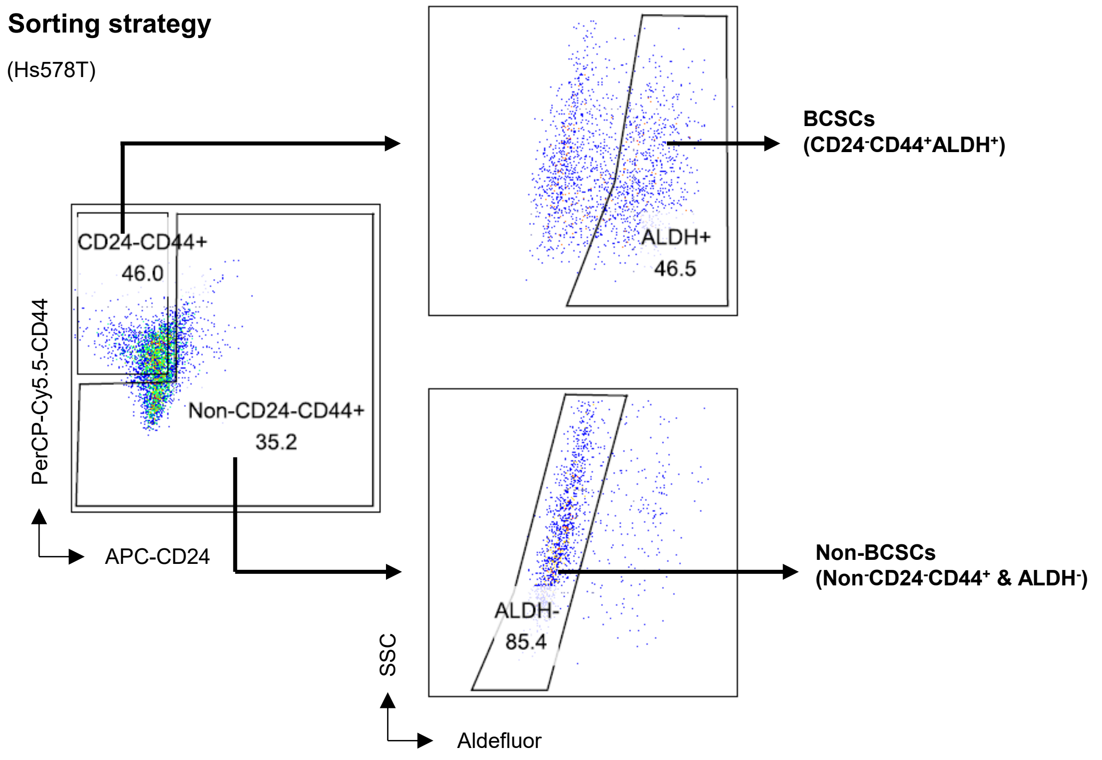

Supplemental Figure 2. Biomarker-based strategy for sorting cancer stem cells (CSCs) in TNBC cells. Flow cytometry-based sorting strategy using established BCSC markers ($CD24^{-}CD44^{+}$ and $ALDH^{+}$) to isolate BCSC and non-BCSC populations from TNBC cell lines for downstream analysis.

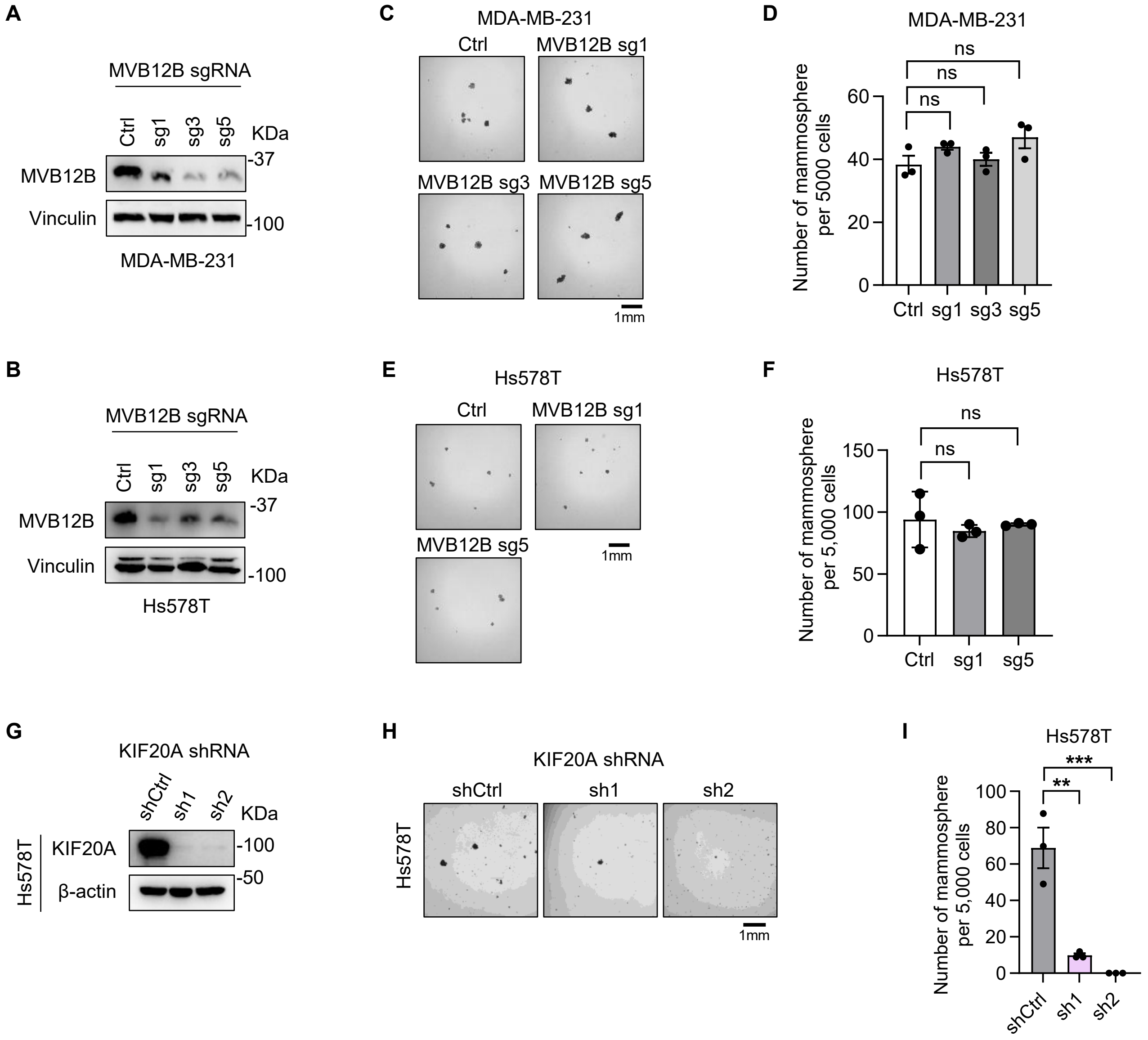

Supplemental Figure 3. Validation of MVB12B and KIF20A depletion in regulating BSSC activity in TNBC. (**A-B**) Immunoblot analysis of MVB12B knockdown efficiency in MDA-MB-231 (**A**) and Hs578T (**B**) cells infected with lentivirus encoding control or MVB12B sgRNAs. (**C-F**) Mammosphere formation assay and corresponding quantifications in MD-MB-231 (**C** and **D**) and Hs578T (**E** and **F**) cells. n=3. (**G-I**) Immunoblot analysis (**G**) mammosphere formation assay (**H**) and corresponding quantifications (**I**) in Hs578T infected with lentivirus encoding control or KIF20A shRNAs. n=3. Data represent mean ± SEM. Statistical analyses were conducted by one-way ANOVA with Dunnett's test (**D, F** and **I**). **p< 0.01, ****p< 0.0001, ns, not significant.

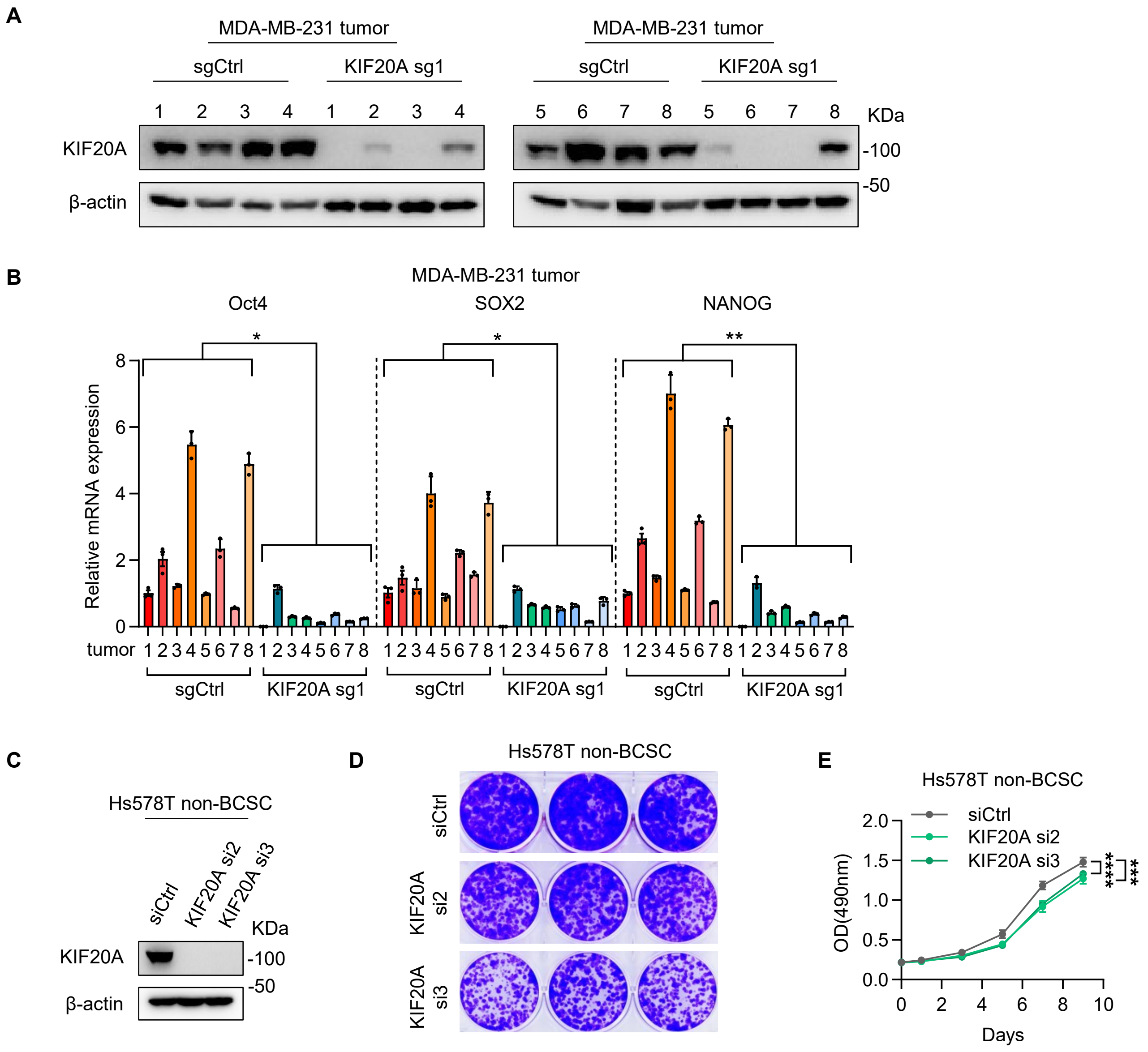

Supplemental Figure 4. Examination of KIF20A depletion in cancer stem cell marker changes and non-BCSC proliferation. (**A**) Immunoblot analysis of KIF20A in tumors infected with lentivirus encoding control or KIF20A sgRNA1. (**B**) qRT-PCR of cancer stem cell markers in control or KIF20A depleted tumors. n=3 (**C**) Immunoblot analysis of KIF20A in Hs587T non-BCSCs transfected with nontargeting control siRNA (siCtrl) or KIF20A siRNAs. (**D-E**) 2D colony formation assay (**D**) and MTS proliferation assay (**E**) of Hs587T non-BCSCs transfected with nontargeting control siRNA (siCtrl) or KIF20A siRNAs. n=3. Data represent mean ± SEM. Statistical analyses were conducted by unpaired 2-tailed Student's t-test (**B**) and two-way ANOVA with Dunnett's test (**E**). *p< 0.05, **p< 0.01, ***p< 0.001, ****p< 0.0001.

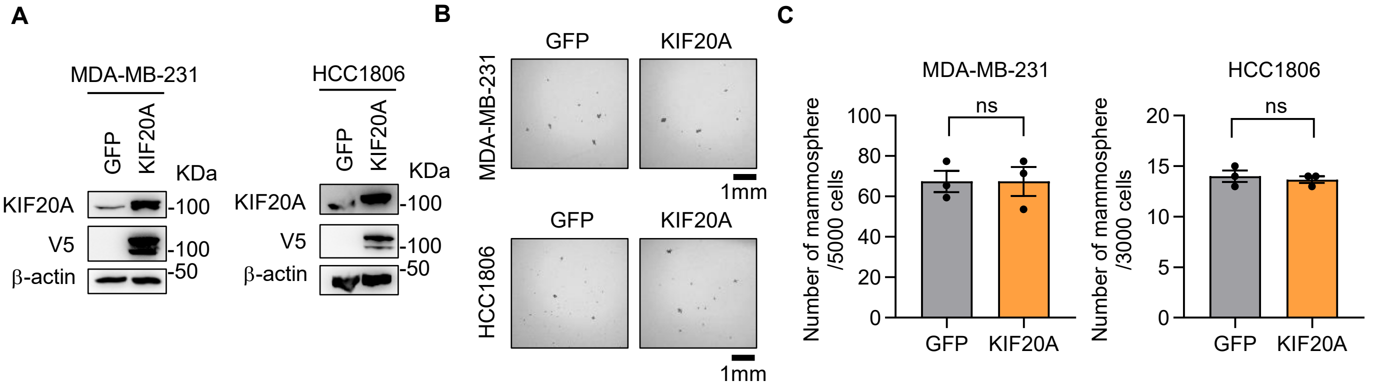

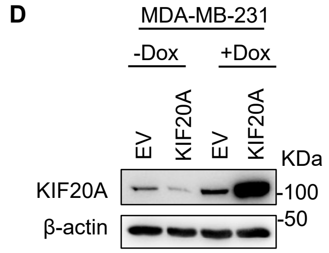

E

# MDA-MB-231 injected		5000	1000	500	200	40	Frequency	p-value
# Mice with tumors	pINDUCER20-EV	5/5	5/5	5/5	4/5	1/5	1:126	
	pINDUCER20-KIF20A	5/5	5/5	5/5	5/5	1/5	1:81	0.483

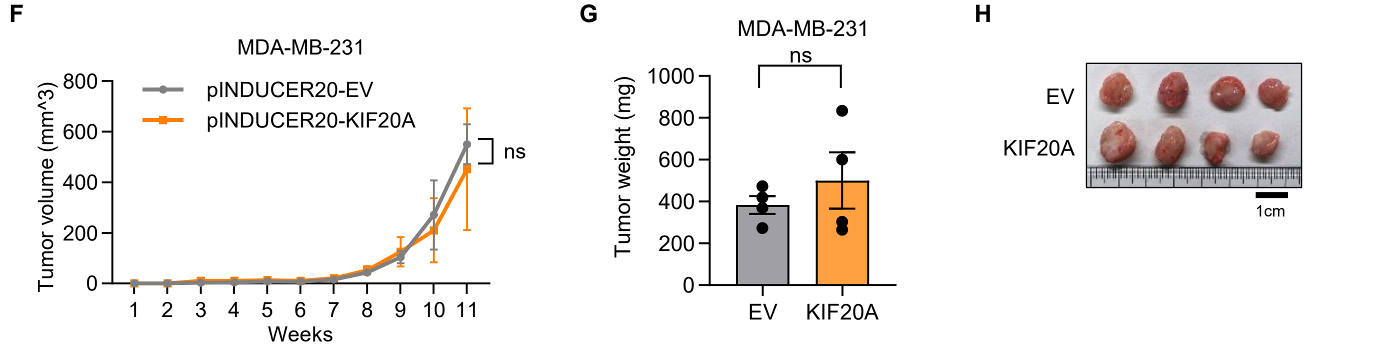

Supplemental Figure 5. Effect of KIF20A overexpression on BCSC self-renewal and TNBC tumor initiation. (**A-C**) Immunoblot analysis (**A**) corresponding mammosphere formation assay (**B**), and number of mammosphere of indicated TNBC cells expressing GFP or KIF20A. n=3. (**D-H**) Immunoblot analysis (**D**), limiting dilution assay of tumor initiating (**E**), tumor growth (**F**), tumor weight after dissection (**G**), and image of tumors (**H**) in MDA-MB-231 cells expressing control empty vector (EV) or KIF20A. n=5. Data represent mean ± SEM. Statistical analyses were conducted by chi-square test (**E**), 2-tailed Student's t-test (**C, G**) or 2-way ANOVA (**F**). ns, not significant.

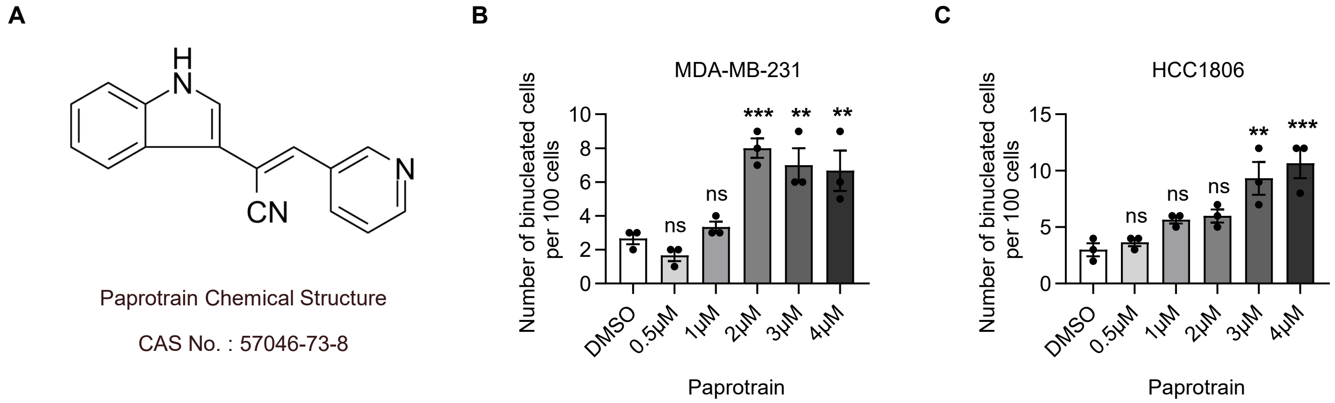

Supplemental Figure 6. Structure information of Paprotrain and its inhibitory effect in TNBC cells. (**A**) Chemical structure of paprotrain. (**B-C**) Quantification of the binucleated cells after two days treatment of paprotrain in MDA-MB-231 (**B**) and HCC1806 (**C**). n=3. Data represent mean ± SEM. Statistical analyses were conducted by one-way ANOVA with Dunnett's test (**B, C**). **p< 0.01, ***p< 0.001, ns, not significant.

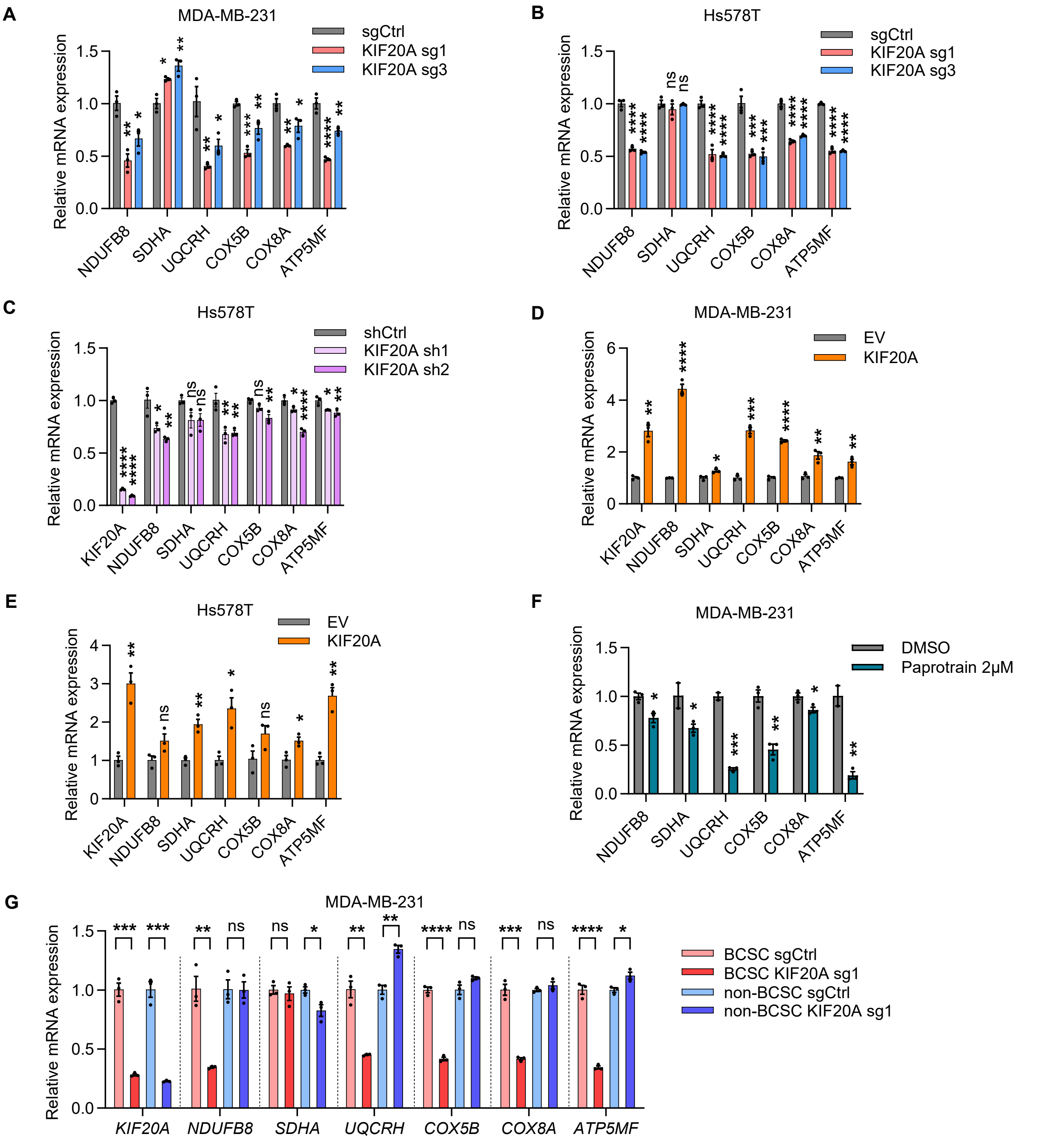

Supplemental Figure 7. KIF20A regulates OXPHOS gene expression. (**A-B**) qRT-PCR analysis of OXPHOS genes in MDA-MB-231 (**A**) and Hs578T (**B**) cells with KIF20A depletion by indicated sgRNAs. n=3. (**C**) qRT-PCR analysis of OXPHOS genes in Hs578T cells transfected with either control shRNA or KIF20A shRNA. n=3. (**D-E**) qRT-PCR analysis of OXPHOS genes in MDA-MB-231 (**D**) and Hs578T (**E**) cells with KIF20A overexpression. n=3. (**F**) qRT-PCR analysis of OXPHOS genes in MDA-MB-231 cells treated with either DMSO or 2 uM paprotrain. n=3. (**G**) qRT-PCR analysis of OXPHOS genes in sorted BCSC or non-BCSC cells from MDA-MB-231 expressing control or KIF20A sgRNA. n=3. Data represent mean ± SEM. Statistical analyses were conducted by one-way ANOVA with Dunnett's test (**A-C**) or 2-tailed Student's t-test (**D-G**). *p< 0.05, **p< 0.01, ***p< 0.001, ****p< 0.0001, ns, not significant.

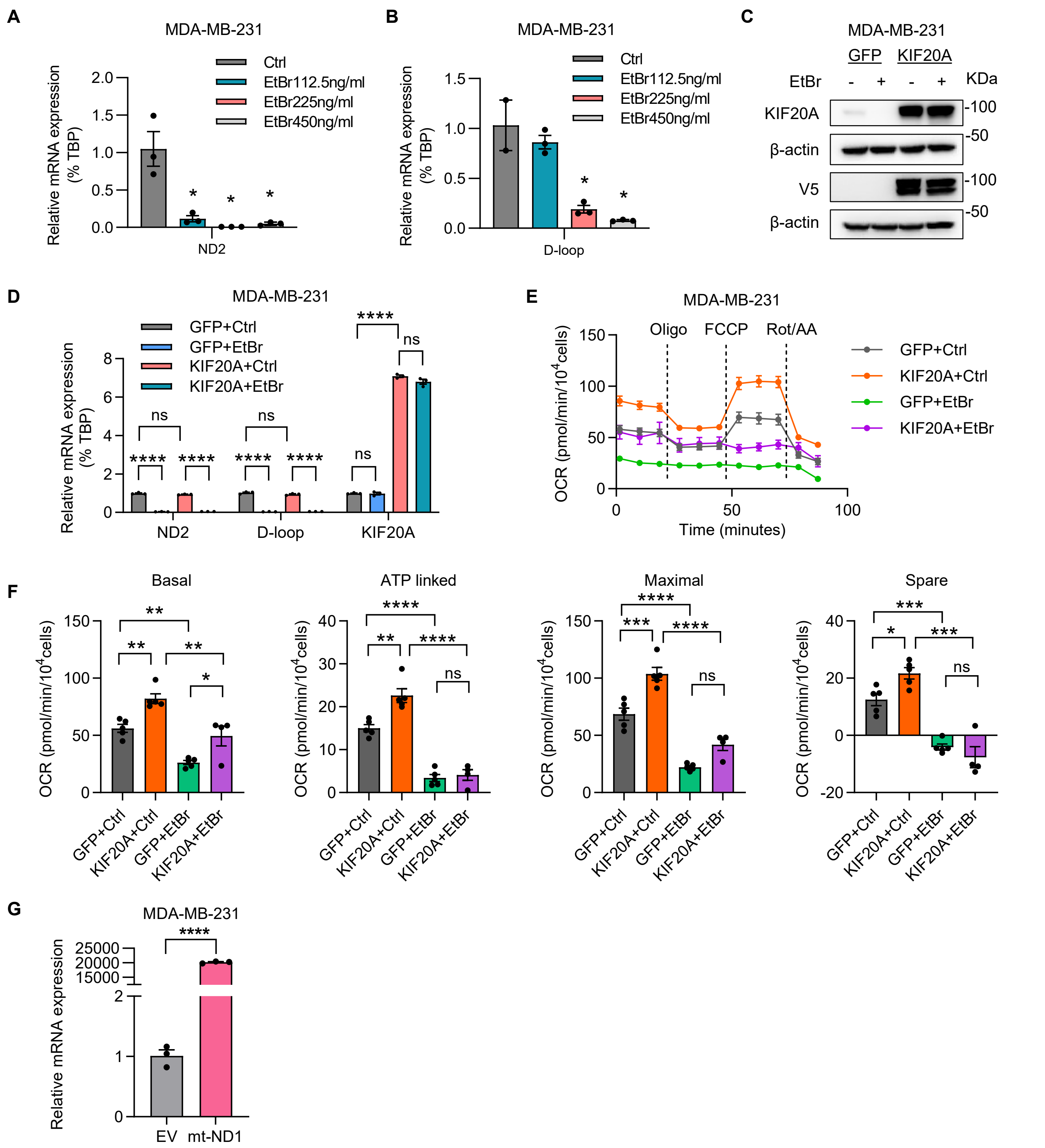

Supplemental Figure 8. Mitochondrial DNA depletion eliminates KIF20A's effect on OXPHOS. (**A-B**) qRT-PCR analysis of *ND2* (**A**) and *D-loop* (**B**) in MDA-MB-231 cells treated either DMSO or EtBr. n=3 (**C-F**) Immunoblot (**C**), qRT-PCR analysis of *ND2* and *D-loop* (n=3) (**D**), and measurement of OCR and quantifications (n=5) (**E** and **F**) of GFP or KIF20A overexpressed MDA-MB-231 cells treated either DMSO or EtBr. (**G**) qRT-PCR analysis of mt-ND1 expression in MDA-MB-231 cells. n=3. Data represent mean ± SEM. Statistical analyses were conducted by one-way ANOVA with Dunnett's test (**A** and **B**), two-way ANOVA with Dunnett's test (**D** and **F**), or 2-tailed Student's t-test (**G**). *p< 0.05, **p< 0.01, ***p< 0.001, ****p< 0.0001, ns, not significant.

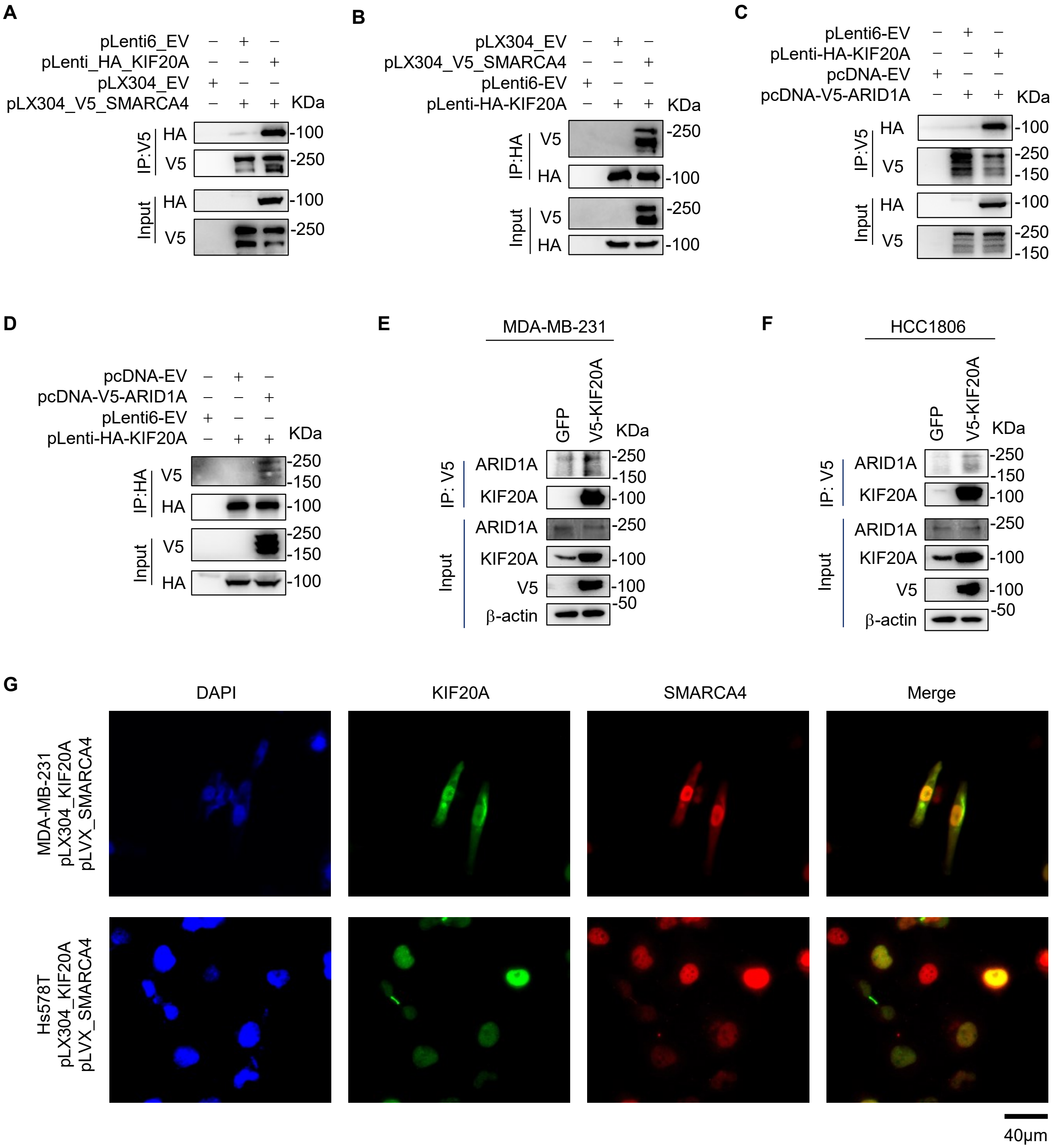

Supplemental Figure 9. Validation of the interaction and co-localization between KIF20A and IP-MS identified candidates. (**A-B**) Co-IP of exogenous HA-tagged KIF20A and exogenous V5-tagged SMARCA4 with V5 beads in TNBC cells by IP with V5 beads (**A**) or HA beads (**B**). (**C-D**) Co-IP of exogenous HA-tagged KIF20A and exogenous V5-tagged ARID1A by IP with V5 beads (**C**) or HA beads (**D**). (**E-F**) Co-IP of exogenous HA-tagged KIF20A and endogenous ARID1A in MDA-MB-231 (**E**) or in HCC1806 (**F**) cells. (**G**) Immunofluorescence (IF) staining of ectopic overexpressed KIF20A and SMARCA4 in MDA-MB-231 or Hs587T cell lines. Scale bar, 40μm.

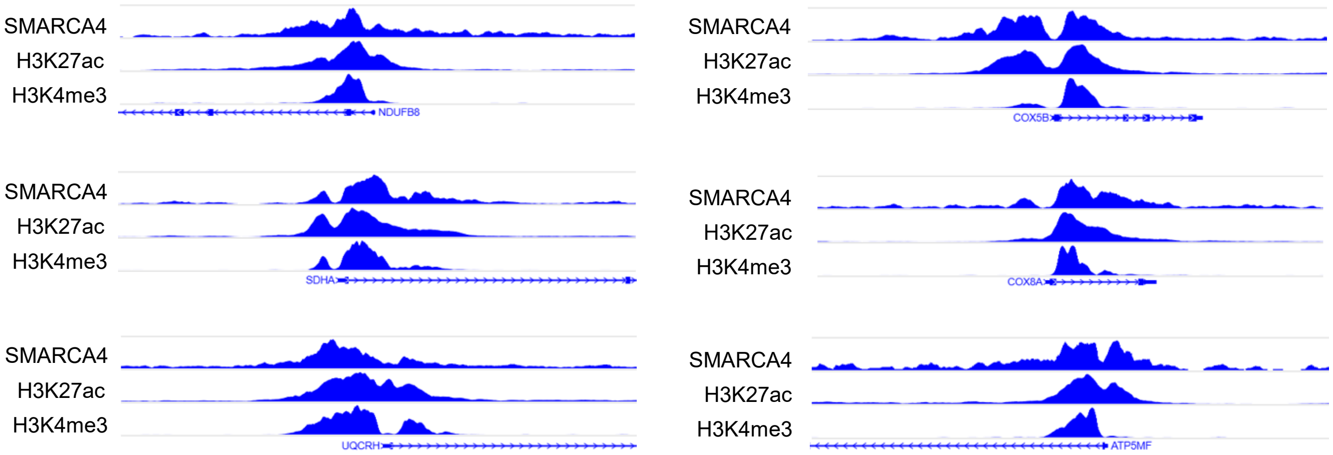

Supplemental Figure 10. SMARCA4 binds to the promotor region of OXPHOS genes. Enrichment of SMARCA4 binding peaks at the promoter regions of selected OXPHOS genes based on previously published ChIP-seq data in MDA-MB-231 cells (Zhou et al., PNAS. 2022;119(39):e2117988119).
