# Supplementary figures and images for: Targeting kinesin family member 20A sensitizes stem-like triple-negative breast cancer cells to standard chemotherapy
Source: J Clin Invest. 2025 Dec 15;135(24):e182394. doi: 10.1172/JCI182394 (PMC12700555; doi:10.1172/JCI182394)

Fig.3A

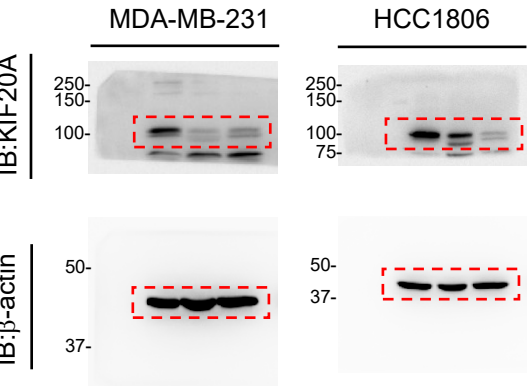

Fig.3D

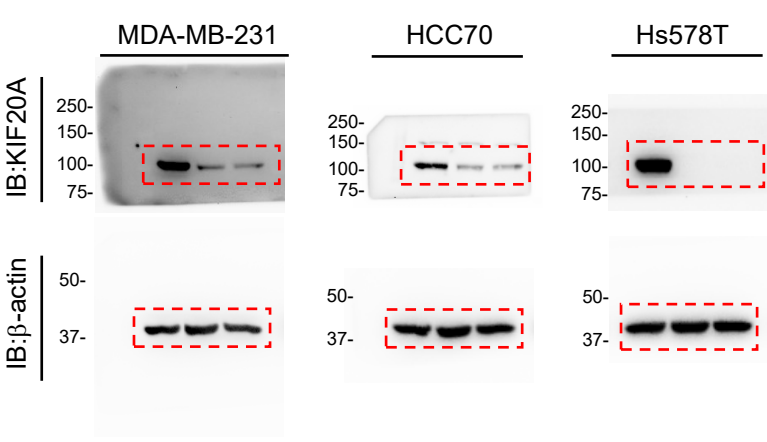

Fig.3I

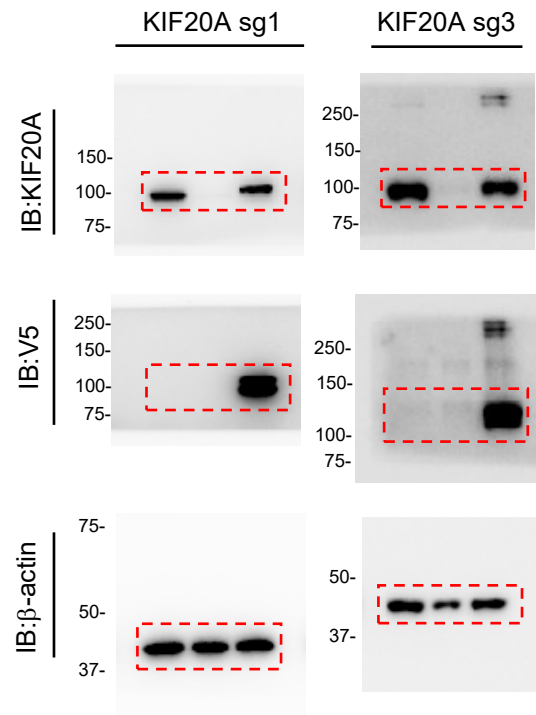

Fig.8C

IB:KIF20A

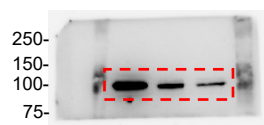

IB:β-actin

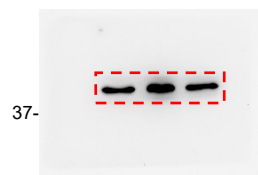

Fig.8H

IB:KIF20A

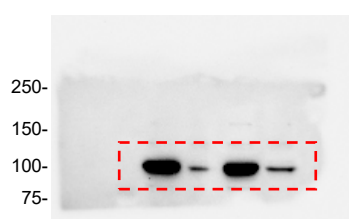

IB:β-actin

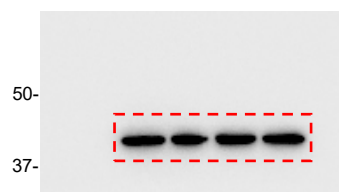

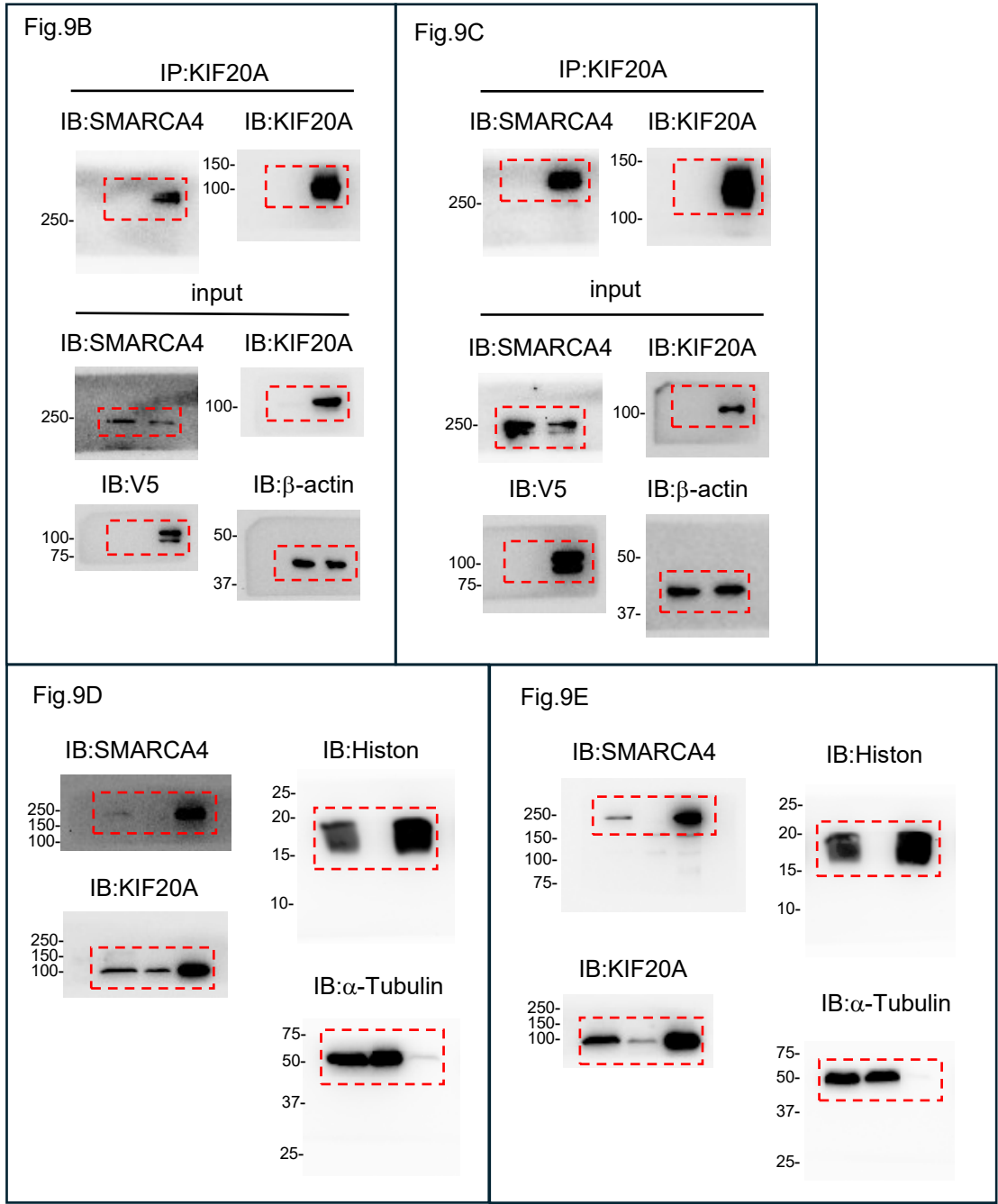

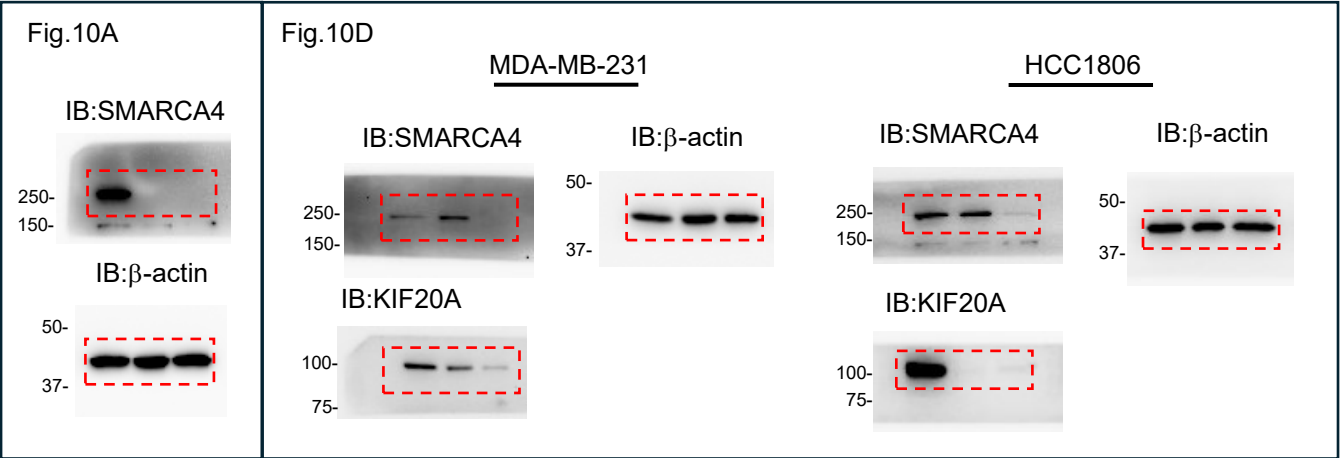

Fig.S3A

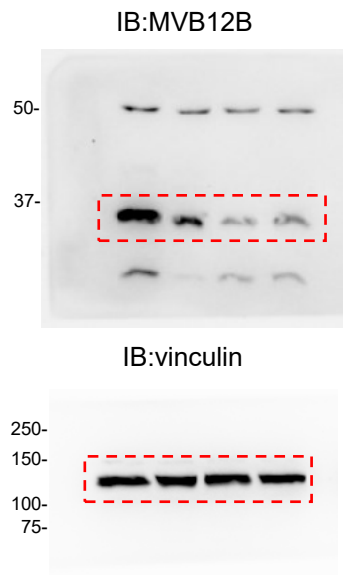

Fig.S3B

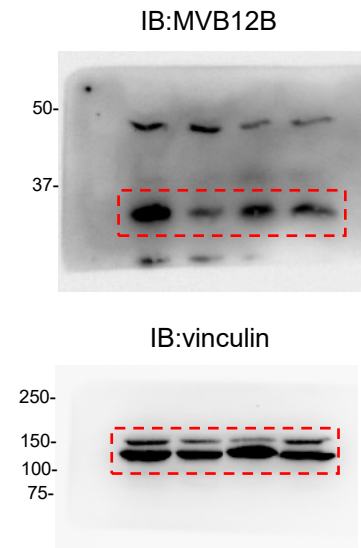

Fig.S3G

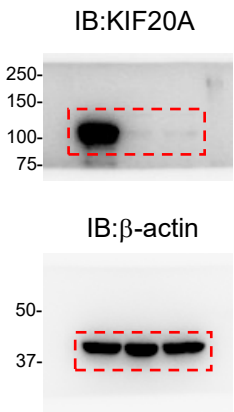

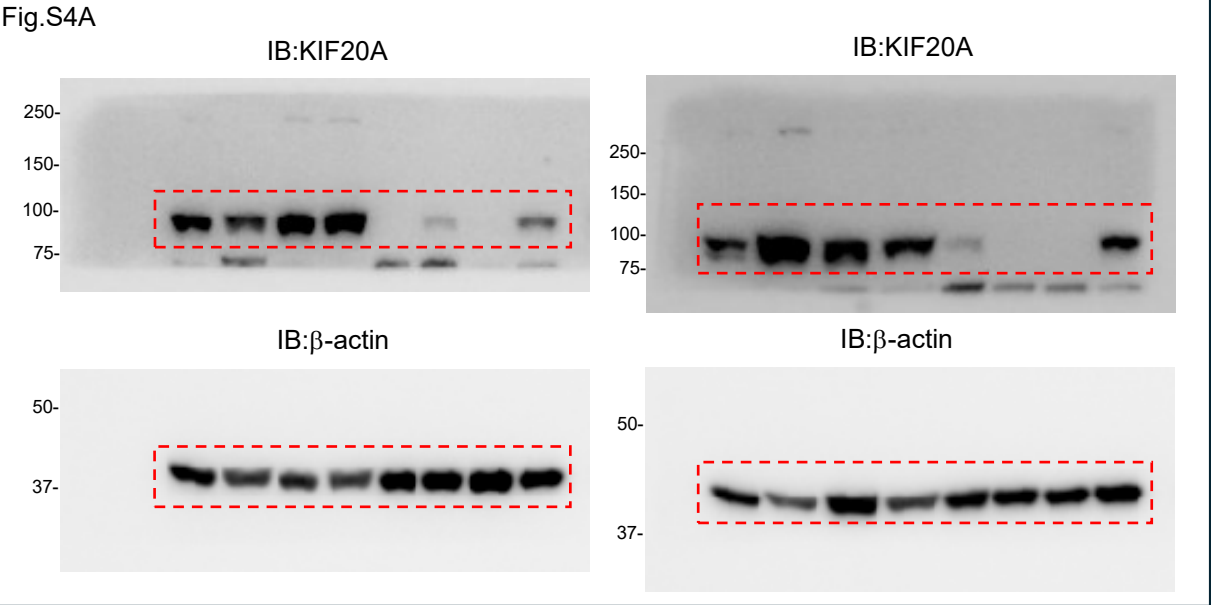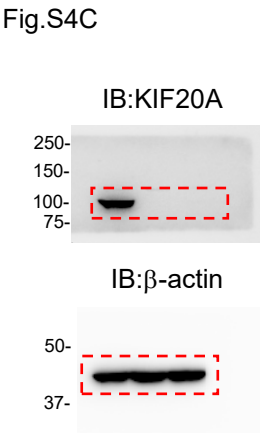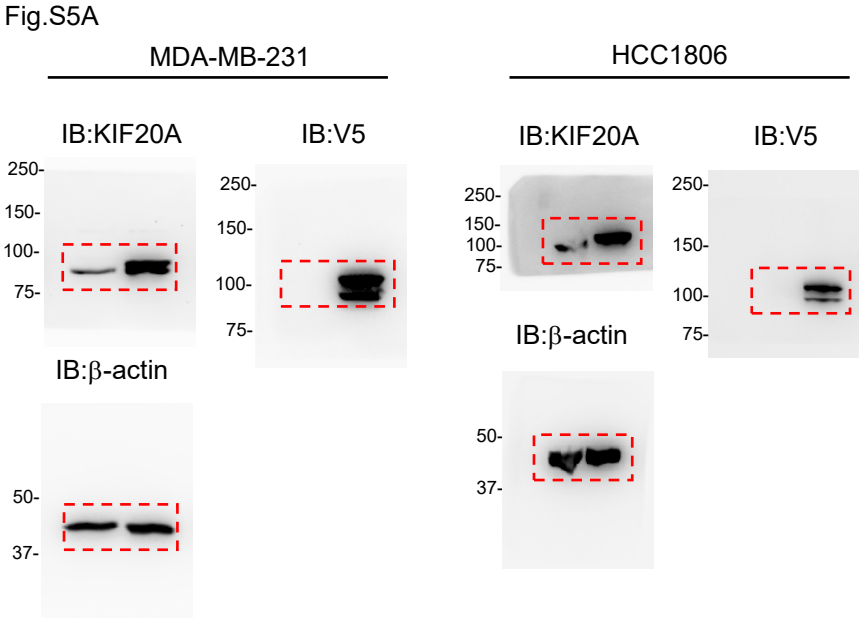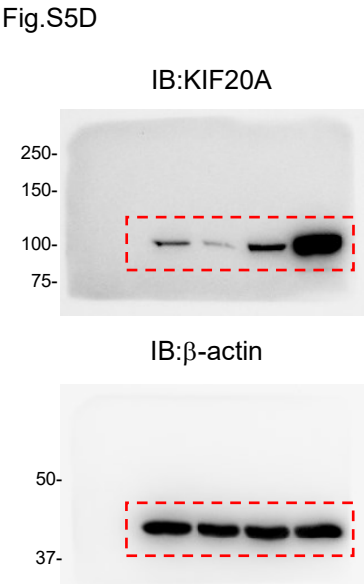

Fig.S8C

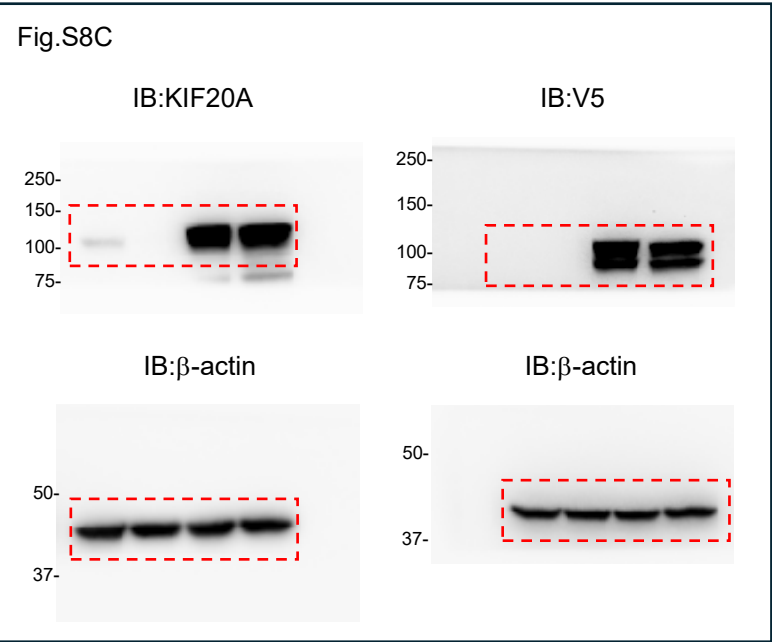

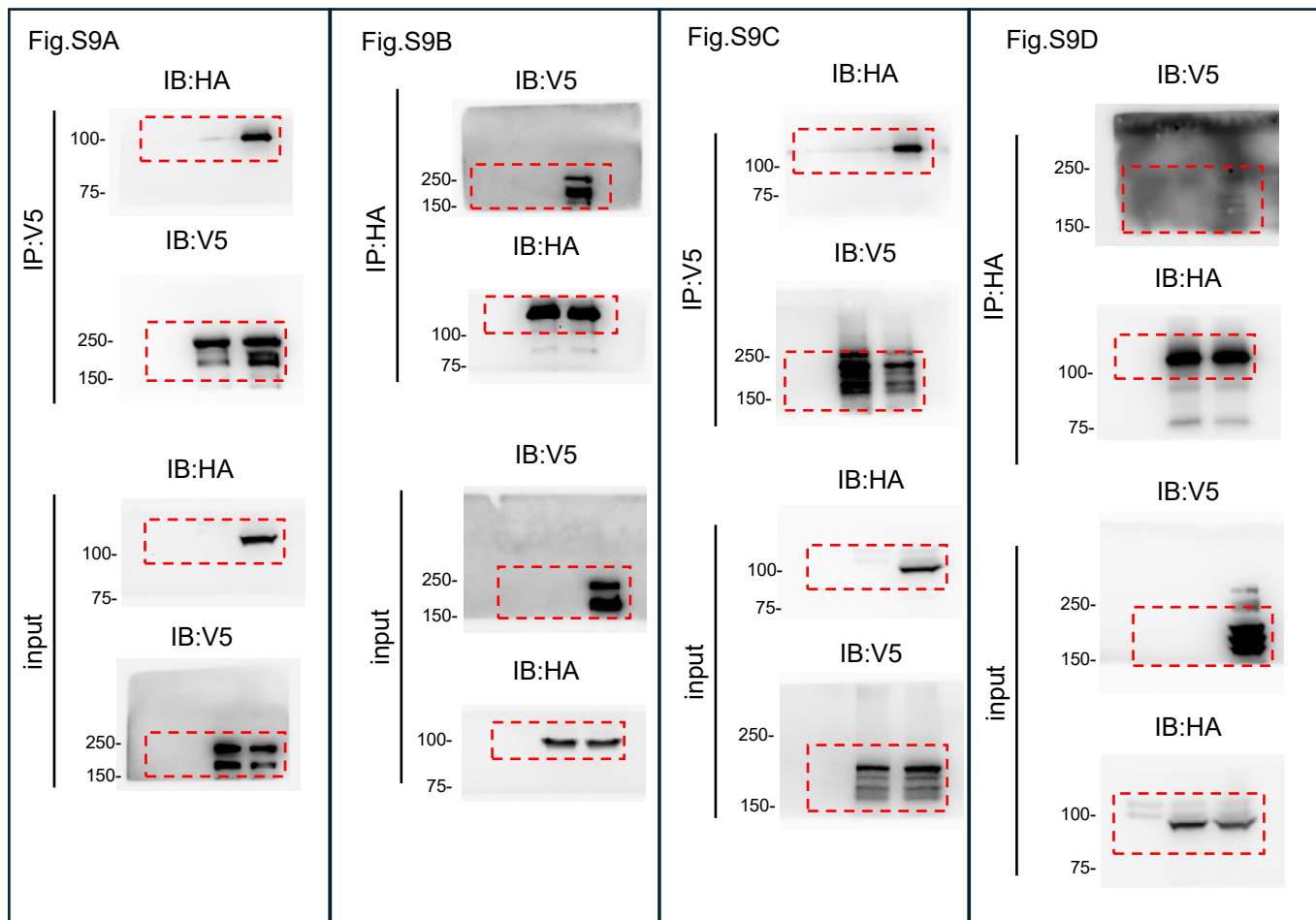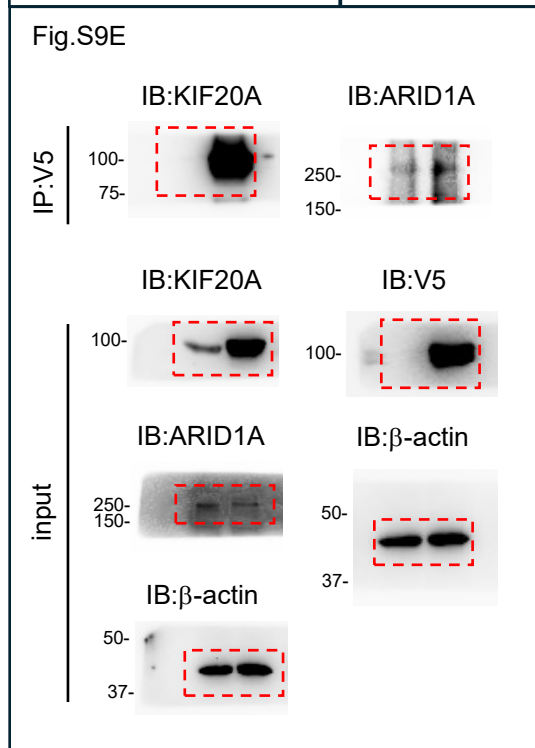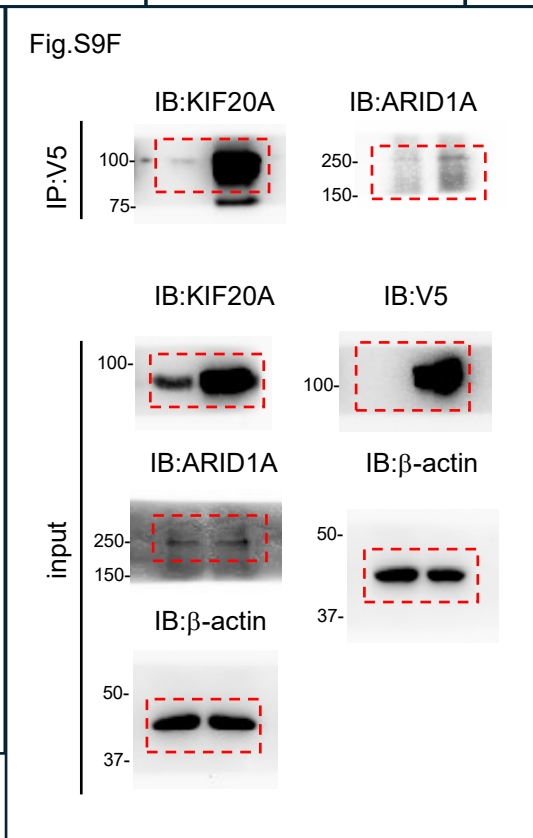

Supplement: Unedited blot and gel images [file jci-135-182394-s099.pdf]
